# Does Ethnicity Affect Ever Migrating and the Number of Migrations? The Case of Indonesia

**DOI:** 10.1007/s10680-023-09694-z

**Published:** 2024-01-30

**Authors:** Elda Luciana Pardede, Viktor Andreas Venhorst

**Affiliations:** 1 Population Research Centre, Faculty of Spatial Sciences, University of Groningen, Groningen, The Netherlands; 2 Master’s Programme in Population and Labour Economics, Department of Economics, Faculty of Economics and Business, Universitas Indonesia, Nathanael Iskandar Building, Lembaga Demografi, 3rd Floor, Kampus UI, 16424 Depok, Jawa Barat Indonesia; 3 Department of Economic Geography, Faculty of Spatial Sciences, University of Groningen, PO Box 800, 9700 AD Groningen, The Netherlands

**Keywords:** Internal migration, Number of migrations, Migration across the lifespan, Ethnicity, Indonesia, Indonesia family life survey

## Abstract

This paper is the first to examine to what extent ethnicity affects ever migrating and the number of migrations across the lifespan for the case of internal migration in Indonesia. We use all five waves of the Indonesia Family Life Survey (IFLS) to study migration behaviour of respondents belonging to some of the largest ethnic groups in Indonesia. Our logistic regression results show that the Minangkabau, Betawi, Madurese, Balinese, Buginese and Makassarese, and Sasak, Bima and Dompu are less likely to ever migrate than the Javanese. Using only migrants and controlling for the first migration and other characteristics, truncated negative binomial regression results show that, in comparison with the Javanese, the Minangkabau and Banjarese have a higher expected number of migrations while the numbers are lower for the Betawi and Balinese. Thus, ethnicity contributes to ever migrating as well as the number of migrations, but we find that the differences between the ethnic groups diminish for the latter. These results also point out that a higher likelihood of ever migrating does not always correspond with a higher number of migrations, highlighting the importance of studying migration count to complement the study of migration as a one-time event.

**Supplementary Information:**

The online version contains supplementary material available at 10.1007/s10680-023-09694-z.

## Introduction

It has been demonstrated that ethnicity is associated with migration in connection to the notion of social capital, referring to the social networks created by the ethnic groups (Reynolds, 2010). Ethnicity is also connected to migration through ethnic capital, defined as an externality from one’s ethnic environment that contributes to one’s human capital accumulation (Borjas, 1992). In this paper, we use these concepts to conceptualise persisting migration behaviour of an ethnic group for the case of Indonesia. Assessing the relationship between ethnicity and migration in Indonesia provides an excellent opportunity to study this issue in the context of a multi-ethnic country because Indonesia is the largest archipelago in the world that hosts about 600 ethnic groups (Ananta, Arifin, Hasbullah et al., 2015), which can be classified into 31 larger ethnic groups (Badan Pusat Statistik [BPS], 2011).^1^ In addition, as will be elaborated in this paper, the relationship between ethnicity and migration in the Indonesian context needs to cover the geographical location and historical processes, such as the colonial period, that have shaped, and continue to shape, the migration behaviour of ethnic groups.

The homes of these ethnic groups are some of Indonesia’s largest islands from west to east^2^: (1) Sumatra: Batak, Minangkabau, Malay, and South Sumatra^3^; (2) Java: Bantenese, Betawi, Sundanese, Cirebonese, Javanese, and Madurese (the island of Madura, the eastern part of Java); (3) Bali: Balinese; (4) Kalimantan: Banjarese; (5) Lombok and its adjacent island Sumbawa: Sasak, Bima, and Dompu; and (6) Sulawesi: Buginese and Makassarese. Islands outside Java were usually referred to as the ‘Outer Islands’ (Hugo, 2006; Tirtosudarmo, 2009).

One of the rationales for studying ethnicity and migration in Indonesia is that intergroup contacts due to migration can lead to various impacts. According to Bazzi et al. (2019), interethnic contacts may foster integration and assimilation that can affect social capital and the provision of public goods. For instance, interethnic groups’ contacts may result in interethnic marriage (exogamy). Ethnic exogamy may lessen ethnic boundaries (see Utomo & McDonald, 2021) and thus be a part of an assimilation process (Rangkuti, 2016) that potentially fosters further different group integration (Bazzi et al., 2019).

On the other hand, migration of different ethnic groups to the same location can lead to polarisation that may reduce social capital, hamper development, and even increase the likelihood of ethnic conflicts (Bazzi et al., 2019). These conflicts may be triggered by the fact that despite Indonesia’s decades of rapid economic development, inequalities among ethnic groups exist. Some, however, attribute inequality in Indonesia more to urban–rural differences (Suryadarma, Widyanti, Suryahadi, et al., 2012) or regional differences (Muttaqien, Sologon, & O'Donoghue, 2018) than ethnicity. Still, inequalities exist between migrant and non-migrant ethnic groups (Jones et al., 2016) and thus interethnic contacts due to migration may create an anti-migrant sentiment and trigger social conflicts. Violent outbreaks among migrant and non-migrant or ‘place-of-origin’ ethnic groups (Van Klinken, 2003) have been recorded in Indonesia (De Jonge & Nooteboom, 2006; Tirtosudarmo, 2009).

Secondly, the relationship between ethnicity and migration is highly relevant for Indonesia, given its vast ethnic diversity and developing economy. The development (modernisation) process is usually accompanied by the growth of population mobility in ‘definite, patterned regularities’, according to the mobility transition hypothesis (Zelinsky, 1971, p. 221). De Haas (2010) further expands this hypothesis by the notion that development affects migration (e.g. via education and information) through migration capabilities—various capitals that can be mobilised to support migration—and migration aspirations—inclinations to migrate. Due to undergoing varying stages of regional development, and historical and demographic processes, different ethnic groups in Indonesia may have diverse levels of migration capabilities and aspirations, which can lead to heterogenous patterns of mobility at any single point in time (Skeldon, 1992).

It is not clear, however, to what extent differences in migration behaviour between ethnic groups are related to socioeconomic and demographic differences between these groups or, instead, are inherent to the ethnic group as such. Despite its significance, studies on the relationship between ethnicity and migration for the case of Indonesia are lacking. Two issues lay at the root of this.

First of all, a cause has been the lack of large-scale, nationally coverage data on ethnicity. Starting from the 1961 Census, for the sake of national unity, the recording of ethnicity was restricted by the government of Indonesia (Ananta, et al., 2015). Earlier, the results of the 1930 Census conducted by the Dutch in Indonesia demonstrated that the intensity of migration^4^ differs by ethnicity (Naim, 1973). For a substantial period, this census was the only basis to consider that some ethnic groups in Indonesia are more likely to migrate than others. Afterwards, studies regarding ethnicity and migration are derived using the birth province information (e.g. Castles, 1967). Fortunately, after the political reformation following the downfall of the Suharto regime in 1998 (Firman, 2004), the Indonesian population’s ethnicity has been re-recorded starting from the 2000 Census. Since then, various studies regarding ethnicity have been conducted using the census (Ananta et al., 2015; Auwalin, 2020; Bazzi et al., 2019; Jones et al., 2016; Rangkuti, 2016; Utomo & McDonald, 2021). The Indonesia Family Life Survey (IFLS) also started to collect data on ethnicity in 2000, covering some of the largest ethnic groups in Indonesia. The Indonesia Family Life Survey (IFLS) also started to collect data on ethnicity in 2000, covering some of the largest ethnic groups in Indonesia.

Based on the IFLS and 2000 and 2010 Censuses, Auwalin (2020) conducted a study on ethnicity and migration. He operationalised migration as moving out of one’s community (defined by the boundaries of an enumeration area)^5^ between two consecutive IFLS waves of 1993, 1997, 2000, and 2007. Auwalin categorised the ethnic groups using the Censuses as high or low migration-rate ethnic groups. He finds that belonging to a high migration-rate ethnic group is positively related to the probability of migrating. However, its effect is lessened when this group is the local majority. Our study adds to this by using ethnicity as the primary explanatory variable instead of classifying ethnicity into having high or low migration rates.

Secondly, our insight into the relationship between ethnicity and migration is limited as migration is often assessed as a one-time event (DaVanzo, 1983). For example, a recent study using the IFLS by Pardede, McCann, and Venhorst (2020) analysed the effects of individual and location characteristics on migrations that were measured by the change of location between two consecutive waves, whether across sub-districts, districts, or provinces. While studies have covered the topic of repeat or return migration (DaVanzo, 1983; Grant & Vanderkamp, 1986), migration is rarely assessed across the lifespan although the IFLS enables us to study migration across the lifespan and the number of migrations as it records every migration since age 12. We therefore argue that studying what affects the number of migrations across the lifespan among migrants contributes to a better understanding of the differences between migration behaviour of ethnic groups. Particularly, Morrison (1971) argues that observed population mobility rates are determined by the relative importance of the mobile segment of the population (the same individuals who moved repeatedly) relative to the stable segment of the population (non-movers and many people undertaking one move). Furthermore, as studies analysing the whole trajectory are still underdeveloped (Wingens, de Valk, Windzio, & Aybek, 2011), including migration, studying migration across the lifespan can contribute to studying the trajectories.

This study, therefore, aims to assess how ethnicity, measured by self-reported ethnicity, affects lifespan internal migration behaviour. We measure migration behaviour by first considering whether the individual has ever migrated across the boundary of a village administrative area.^6^ We then consider the number of migrations across the life trajectory for members of various ethnic groups in Indonesia. Our research question is thus formulated as: ‘To what extent does ethnicity affect ever migrating and the number of migrations, over and above individual demographic and socioeconomic characteristics?’ This study is the first to do so by fully employing the migration history information in the IFLS recorded for participants starting at the age of 12 up to the end of observation.

## Ethnicity and Migration

We derive four general principles from the literature that help shape our thinking on the association between ethnicity and migration and divided them into two parts. The first part is related to what ethnicity can represent concerning migration: (1) migration as a part of culture (Boyle et al., 1998) that can be viewed as ethnic capital (Borjas, 1992) and (2) ethnicity as social capital (Reynolds, 2010) such as segmented migration networks and labour markets (Skeldon, 1990) of each ethnic group. The second part consists of two principles that are related to the context of Indonesia: (3) the impact of colonialism in shaping migrations (Skeldon, 1990), particularly in Indonesia (Hugo, 1978; Tirtosudarmo, 2009); and (4) the geographical locations of the ethnic groups: whether their home regions are more in the centre of economic, administrative, demographic activities or the periphery (Titus, 1978). We use these principles to shed light on processes that could be historical, cultural or contextual, that shape the differences between the mobility of ethnic groups today. As will be apparent, these principles are, to some extent, intertwined. As a result, we cannot identify which of these four causes lays at the root of these results. It was demonstrated using the 1930 Census that the ethnic groups such as the Batak, Minangkabau, Banjarese, and Buginese, to have higher migration intensities, while ethnic groups such as the Malay, Javanese, Sundanese, Madurese, Makassarese, Balinese and Sasak have lower migration intensities (Naim, 1973). Also, according to the 2000 and 2010 Census (Auwalin, 2020), three ethnic groups have consistently higher migration rates: the Batak, Minangkabau, and Buginese.^7^ While changes in the migration rates occurred across these censuses,^8^ our starting hypothesis is that these groups have a higher likelihood of ever migrating and a higher number of migrations than the others. In what follows, we illustrate the four general principles for these and some of the larger ethnic groups recorded in the IFLS.

### Ethnicity and Migration: Some Theoretical Explanations

#### Migration Behaviour as a Part of Culture and Ethnic Capital

In many cultures, migration is an integral part of people's lives. The selectivity of migration may generate a culture of migration that reproduces migration experiences that are made relatively permanent experiences in the life course of members of societal groups, which may even be highly valued by people within these groups (Boyle et al., 1998). Extending the concept of ethnic capital (Borjas, 1992), we presume that one’s ethnic environment provides a capital that one can draw upon, in the form of skills related to migration behaviour, shaped by the ethnic group’s migration experiences, external to the individuals. This form of ethnic capital triggers a higher likelihood of migrating of a particular ethnic group than the other.

Among the Minangkabau from West-Sumatra, migration has traditionally been imposed on men because of the weak roles they have in their mothers’ or their wives’ households in terms of wealth and inheritance due to their matrilineal culture (Naim, 1973). Thus, Minangkabau males, particularly unmarried men, tended to move to earn their livelihoods elsewhere, although migration of the Minangkabau females, many as tied migrants, also occurred. Therefore, migration has long been established as a part of their culture (Chadwick, 1991; Naim, 1973). Likewise, the Buginese from South Sulawesi province, who have been famous as seafarers (Van Klinken, 2003), travelled as traders and settlers to various parts of Indonesia and even to the Malay peninsula, noted back since the late seventeenth century. Migration of the Buginese is closely linked to their view that *pasompe* (migrant or wanderer), a nameless culture-hero, is an attribute of their society. Their society highly values those ‘who have travelled across the sea to seek their fortunes’ (Lineton, 1975, p. 174). Therefore, we expect that the members of ethnic groups with migration as a part of culture have a higher likelihood of ever migrating and moving more often than the members of other ethnic groups as they provide ethnic capital that promotes migration.

#### Ethnically Segmented Migration Networks and Labour Markets

Ethnicity is also associated with migration as it is connected to social capital, acquired through social relations with others (Riedel, 2015), established by each ethnic group, such as migration networks and labour markets. The networks created by these groups tend to create further migration from those particular groups (Reynolds, 2010). In Indonesia, some ethnic groups established themselves in concentrated areas in a wider destination (Hugo, 1978; Jones, et al., 2016). These established networks and social contacts may trigger specialisation in particular occupations of people from a particular area (Skeldon, 1990).^9^

The Buginese migrants from South Sulawesi established chain migrations, usually by inviting or funding some of their relatives or fellow villagers to come to the destinations and created new homes (Lineton, 1975). Networks can also be created in the forms of ethnic associations in the destinations such as by the Minangkabau (Naim, 1973). The Batak usually formed family names (*marga*) associations that can assist new Batak migrants (Bruner, 1972).

Also, networks can be created by reproducing social systems from the home regions elsewhere. For example, the Balinese, who have the lowest intensity of migration according to the 1930 Census (Naim, 1973), have very complex long-established social systems that they use at their home regions to organise their community (*Banjar*), agriculture (*Subak*) and religious (*Pura*) affairs (Geertz, 1967). By reproducing their unique social systems in particular destinations (e.g. in transmigration areas (Roth, 2011) the Balinese may encourage migration of other Balinese to these destinations. The need to reproduce these systems is perhaps a necessity to organise their community lives in the area of destination, given that they are usually ethnic and religious minorities outside their home region as they are mostly Hindu, while Indonesia as a whole is predominantly Muslim.

Because of these networks, people from particular ethnic groups may have also created segmented labour market opportunities for their ethnic group members in some locations that may trigger migration to those locations. Therefore, the segmented labour market opportunities may represent a specific economic opportunity to an ethnic group in a particular location. We presume that people from an ethnic group that tends to occupy jobs that require stable locations will move less often than the others. Trading, for example, may require more movement than working as a civil servant. Those who were famous for moving as traders were the Makassarese and Buginese (Lineton, 1975), the Minangkabau (Chadwick, 1991), and the Madurese, who were also famous for working as temporary labourers (De Jonge & Nooteboom, 2006).

Some of these segmented labour markets were notably created during the colonial era through the impact of Western education. Western education in Indonesia was earlier introduced as a segregated system limited to the (elite) natives. After the introduction of ‘Ethical Policy’ aimed to improve the conditions of the native population, the Dutch launched people’s schools at the village level or collaborated with the schools established by Christian missionaries (Tomagola, 1982). It was partly motivated by the need to provide lower-level skilled workers for the Dutch in Indonesia (Tirtosudarmo, 2009). This education system was established alongside Islamic education (*pesantren*) that had been part of Muslim lives in Indonesia. The ethnic groups that, to a higher degree, embraced Western education in the past enjoyed better employment opportunities in more stable, higher-skilled jobs than those who did not. To some extent, this historical process may also be related to ethnic capital, referring to the average level of skills possessed by the members of an ethnic group (Borjas, 1992). The Minangkabau and the Batak are examples of such ethnic groups (Naim, 1973; Rodenburg, 1997).

Therefore, we expect that the ethnic groups that had established migration networks and segmented labour markets, partly due to the impacts of Western education in the past, tend to have a higher likelihood of migrating than the others. As previously discussed, diverse migration flows might have established these networks and segmented labour opportunities. Some flows were from the lower end of the socio-economic spectrum, such as people with unstable jobs who might have conducted numerous movements out of necessity. Others were from the higher end of the socio-economic spectrum, such as those impacted by Western education.

### Ethnicity and Migration: The Context of Indonesia

#### The Impact of Colonialism in Shaping Migration of Indonesians

Contemporary migration patterns can be understood from their historical backgrounds (Hugo, 2006). The Indonesian archipelago has a long and complex history of migration and settlement since the prehistoric time that has shaped and formed its population’s settlement, language, and ethnic diversity (Tumonggor et al., 2013). Still, Western colonisation is considered to largely influence post-colonial mobility patterns in Indonesia and developing countries in general (Skeldon, 1990). Hugo (2006) maintains that population movement in Indonesia during the colonial time was impacted gradually following the increase in the influence and control of the colonial rule from the sixteenth century, culminating in the imposition of direct colonial rule by the Dutch government in the nineteenth century. Hugo points out some of the effects on migration brought by this imposition such as revolution in transportation availability and patterns, drastic changes in the structure of the economy which created new and different types of job opportunities along with the introduction of wage employment, laws to encourage or discourage particular types of movement, the existence of forced and semi-forced labour schemes, the development of urban centres and migration towards them, and the introduction of primary and, to a lesser extent, secondary schools.

Colonialism, thus, influenced the lives of people who belonged to specific ethnic groups in different ways and was thus shaping their migration behaviour, directly or indirectly. In a direct sense, colonial activities created new migration patterns outside the traditional mobility patterns (Skeldon, 1990) or accentuated these traditional patterns. Indirectly, migration could also be a reaction to these colonial activities.

Labour was recruited for plantations or mining, mainly from the island of Java. Thousands of Javanese and Sundanese workers were transported from Java to Sumatra or the Outer Islands (Hugo, 1978; Tirtosudarmo, 2009), sometimes with some elements of force (Hugo, 2006). These economic activities also generated demand for labour from more nearby areas, which are the home regions of particular ethnic groups. For example, some of the Batak^10^ and also the Minangkabau in Sumatra responded by moving to the east coast of Sumatra for wage-labour or trade opportunities (Chadwick, 1991; Cunningham, 1958; Rodenburg, 1997).

The selection by the colonials of particular locations as the centres of economic activity, while other areas lagged, created inequalities between different groups and thus also played a role in influencing migrations (Hugo, 2006). The creation of urban centres in Java, such as Batavia—the former name of Jakarta Special Region, the provincial capital of Indonesia—Semarang in Central Java, and Surabaya in East Java, triggered migration within Java and from the Outer Islands (Tirtosudarmo, 2009). Preferential recruitments also drove the migration of people from the Outer Islands to Java. The Dutch preferred to recruit the Outer Islands ethnic groups, such as from the eastern part of Indonesia (Hugo, 1978), who had engaged in Western education by the Christian missionaries, mostly Christianised, as functionaries such as soldiers, police, and low-level administrative staff in Java (Hugo, 2006).

Aside from this, the Dutch implemented the resettlement policy after they considered Java Island to be overpopulated in 1905. As a result, people were resettled from Java to the Outer Islands. The government of Indonesia adopted this policy after independence in the programme called 'transmigration' (Nitisastro, 2006).

Migration could also be a reaction to an act of war by the colonialists. For the case of the Banjarese from South Kalimantan, the Banjar war with the Dutch in the mid-nineteenth century (Hawkins, 2000; Hugo, 2006) forced some of the Banjarese to flee from their territories, primarily to their established migration destinations in Sumatra and Malaysia. According to the 1930 Census, many of them resided in the eastern part of Sumatra, Riau and Jambi provinces in Sumatra, even abroad, such as in Malaysia (Naim, 1973).

Hence, the ethnic groups that were most influenced these colonial activities in terms of their likelihood of migrating in the past may have a higher likelihood of migrating than other ethnic groups. It may be in the form of chain migration linkages between the areas or return migration (Hugo, 2006). Thus, we expect these ethnic groups to have a higher likelihood of migrating than the others.

#### The Home Region of Ethnic Groups

The second element that may shape ethnic differentials in migration is the home location, which is the traditional home region of each ethnic group. In Indonesia, Java is the most fertile and was developed more than the rest of Indonesia during the colonial era. This development has been perpetuated by the government of Indonesia after independence (Douglass, 1997; Tirtosudarmo, 2009). Consequently, Java has been the political and economic centre (Titus, 1978). It is the most developed and densely populated island, followed by the island of Sumatra (Firman, 1997), while the rest of Indonesia is lagging.

The consequence of this extent of unequal development between Java and elsewhere within Indonesia is that Java, and the ethnic groups which are dominant there, exhibit high intra-island migration on top of attracting migrants from the Outer Islands (Douglass, 1997; Hugo, 1988; Wajdi, van Wissen, & Mulder, 2015). On the other hand, extractive industries such as mining and plantations in the peripheral locations outside Java did attract people to move to the Outer Islands (Titus, 1978). However, these outflows from Java were small relative to the inflows as well as Java population size (Naim, 1973).

As the migrations of the population in Java have been concentrated more within Java than to the other parts of Indonesia (Hugo, 1988), the local ethnic groups in Java are the majority in their home regions: the Javanese and the Sundanese—two of the majority ethnic groups in Indonesia, and the Bantenese (Ananta et al., 2015). On the other hand, there is an ethnic group in Java Island, the Betawi^11^ from Jakarta, which is not the majority in their home region. Only 28 per cent of Betawi people lived in Jakarta, according to the 2010 Census (Jones et al., 2016). Their moves, however, were mainly to the surrounding areas of Jakarta (Jones, et al., 2016).

It is an intricate task, however, to determine the effects of the home location of the different ethnic groups in Java and outside Java on their migration patterns across the lifespan. In general, Pardede et al., (2020) find that living in Java is mostly associated with a lower probability to migrate than living outside Java. Nevertheless, migrants from outside of Java may tend to move directly to urban centres in Java, mainly as positive selectivity might play a role in migration from farther away (Jones et al., 2016). Such moves may involve more costly transportation, so the migrants tend to move directly rather than repeatedly to reach their intended destination. However, within Java, migrants may be able to move in steps to get to their ultimate destinations. Therefore, we expect that the ethnic groups with home regions from outside Java are more likely to ever migrate than those from Java itself, while the ethnic groups from Java are more likely to move repeatedly thus have a higher number of migrations than those from outside Java.

### Other Characteristics that Determine Ever Migrating and the Number of Migrations

In this section, we distinguish three types of characteristics that are associated with migration across the lifespan (ever migrating and the number of migrations): the individual characteristics, the location of origin (i.e. the place before moving, not necessarily the home region of one’s ethnic group) characteristics, and, only in the analysis of the number of migrations, the first migration characteristics. Some individual characteristics that affect migration are birth cohort, gender, education, and parents’ education as a measure of socioeconomic status. Birth cohort situates individuals in a particular socio-historical process as they progress through their lives (Elder, 1975). Younger cohorts usually face a higher level of development, urbanisation, better transportation and communication facilities than the older cohorts in their lifetime. Thus, we expect the younger cohorts to have a higher likelihood of migrating than the older cohorts. In the context of repeat migration, it is found that males are more mobile than females (Grant & Vanderkamp, 1986). Therefore, we also expect more migration across the lifespan for males. As education may depict one’s tendency to be responsive to opportunities elsewhere and is mostly positively related to repeat migration (DaVanzo, 1981, 1983), education and parents’ education are also expected to be positively related to migration across the lifespan.

Aside from the roles of an ethnic group home region on migration, the locations where the members of an ethnic group are born and raised also affect migration. A longer distance to the urban centres may necessitate migration, while a shorter distance to the urban centres may trigger step migration towards the urban centre. Therefore, the distance of one's place of birth from the capital city can be positively related to ever migrating but negatively related to the number of migrations. According to the region at the beginning of migration trajectories, we also expect differentials in migration: living in Java is expected to be negatively associated with ever migrating (Pardede et al., 2020) while positively related to the number of migrations. Also, the degree of urbanity of the area at the beginning of the migration trajectories may affect migration. As cities or urban centres are considered the ultimate destination of migration (Skeldon, 1990), we expect living in rural areas to be negatively related to ever migrating (Pardede et al., 2020) but positively associated with the number of migrations. By incorporating these location variables, we control that a Javanese born further away from the capital city and raised in a rural area outside Java may face different economic opportunities and levels of development compared with a Javanese born and raised in an urban area on Java island.

We also account for the characteristics of the first migration. We presume that the first migration since the start of the observation is a 'highly consequential' transition and may 'yield a persistent effect' (Wingens et al., 2011, p. 13) on the subsequent migration, and thus the number of migrations across the lifespan. Firstly, we use the age of the first migration. As age describes a stage in an individual's life (Elder, 1975), the age of the first migration marks the timing of the start of migration behaviour across a migration trajectory. First migration at a later age delays the timing of the next migration and thus is negatively associated with the number of migrations. As subsequent migration may depend on the reason for the first migration, a move for work or education may trigger more moves than a move for marriage or family. Moving for family reasons or marriage may create more ties to the location of origin (DaVanzo, 1981; Grant & Vanderkamp, 1986). Consequently, we expect those who move for the first time for education or work to have a higher number of migrations than those who move for marriage or familial reasons.

Lastly, we also expect the length of stay at the destination after the first migration to be negatively associated with the number of migrations. The longer one stays in a location, the more location-specific capital has been formed (DaVanzo, 1981) and the less likely subsequent moves then become. Additional variables to control for exposure and timing, ever migrating abroad to control for the time exposed to the risk of migrating, the year of the first migration to control for periodic shock, the age at the end of observation, and the year of entering the survey will be discussed in the method section.

## Data and Method

**Fig. 1:**
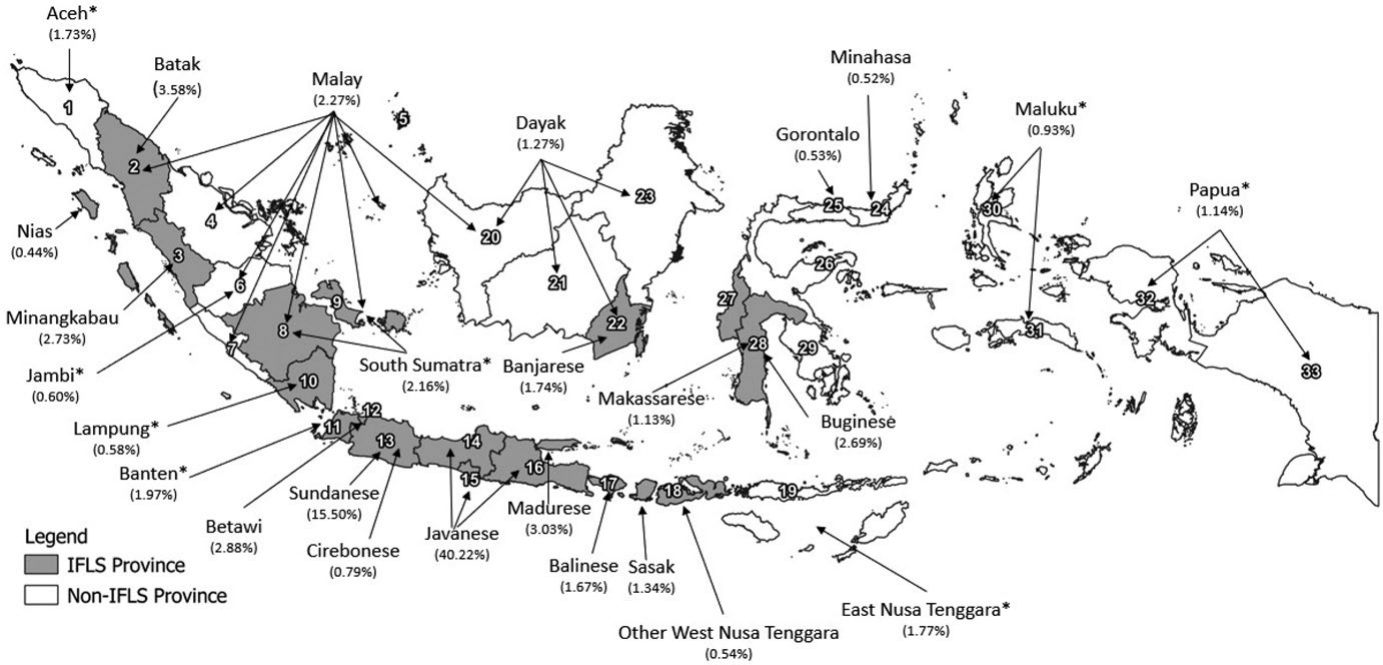
Provinces covered in Indonesia Family Life Survey and the distribution of the largest ethnic groups in Indonesia by the province of origin, Census 2010. *Source:* Calculated from BPS (2011). The map depicts mostly the largest ethnic groups (refer to Appendix Table A.1 for the full table). These are 33 provinces according to the 2010 Census: 1. Aceh, 2. North Sumatra, 3. West Sumatra; 4. Riau; 5. Riau islands; 6. Jambi, 7. Bengkulu, 8. South Sumatra, 9. Bangka Belitung, 10. Lampung, 11. Banten, 12. DKI Jakarta, 13. West Java, 14. Central Java; 15. DI Yogyakarta, 16. East Java, 17. Bali, 18. West Nusa Tenggara, 19. East Nusa Tenggara, 20. West Kalimantan, 21. Central Kalimantan, 22. South Kalimantan, 23. East Kalimantan, 24. North Sulawesi, 25. Gorontalo, 26. Central Sulawesi, 27. South Sulawesi, 28. Southeast Sulawesi, 29. West Sulawesi, 30. Maluku, 31. North Maluku, 32. West Papua, 33. Papua. These provinces used to be one province in 1993: province 4 and 5 (Riau); 8 and 9 (South Sumatra); 11 and 13 (West Java); 24 and 25 (North Sulawesi); 27 and 29 (South Sulawesi); 30 and 31 (Maluku); and 32 and 33 (Irian Jaya). Note: *Includes all ethnic groups from this province. For example, Banten includes the Bantenese and other ethnic groups in Banten province

The Indonesia Family Life Survey (IFLS)^12^ was conducted for the first time in 1993, covering 13 provinces in Indonesia's western and middle parts out of the 27 provinces including Timor Leste (see the map in Fig. 1). The respondents of the first wave were then followed in 1997, 2000, 2007, and 2014. Selected (targeted) respondents were interviewed for detailed information, including migration history (for details, see Frankenberg and Karoly (1995) and Strauss, Witoelar, & Sikoki (2016)).

Overall, the IFLS covers most of the largest ethnic groups, roughly about three-quarters of the population (see detailed discussion in Appendix, Online Resource, Table A.1 and A.2). Targeted respondents were tracked if they still resided in one of these 13 provinces, regardless of whether they moved across those provinces. Consequently, our analysis concentrated on the experience of people who (continued to) reside in these provinces and are more likely to belong to ethnic groups originating from these IFLS provinces. Many smaller ethnic groups, particularly those originating from outside the IFLS provinces who migrated or the descendants of migrants in the IFLS provinces, were treated as a residual category. For example, we grouped a few Maluku people surveyed in South Sulawesi into the residual category ‘others’. In contrast, others from eastern parts of Indonesia, such as East Nusa Tenggara and Papua, are unavailable.

The sample for this study was derived by combining all five IFLS waves. IFLS recorded migration history starting from age 12 of targeted adult respondents who were at least 15 years at the time of the survey. Migration in this survey is defined as moving at least across the village administrative border and staying in the destination for at least six months. We used all respondents with migration trajectories from age 12 until at least age 36. Furthermore, even though moves to and from abroad were recorded, we excluded these migrations as we focus on internal migration and continued exposure in Indonesia is presumed. A respondent who often moves abroad may have fewer migrations across the lifespan than another who only moves internally.

The analysis was conducted as a two-step procedure. The first is an analysis of ever migrating internally across the lifespan using 21,427 respondents (migrants and non-migrants). Next, we analyse the number of internal migrations using 12,161 migrants.

The dependent variables of our regression models are: (1) ever migrating using logistic regression model (Model A) and (2) the number of migrations across the lifespan using truncated negative binomial regression for count outcomes (Model B). Both models were estimated by clustering the standard errors at the household level at the respondent’s year of entry to IFLS. The truncated negative binomial regression models count data, the number of events which occurs during a time interval, for zero-truncated samples. We calculated the exponential value of the estimates to get the expected number of migrations (see Long, 1997; Long & Freese, 2014). The zero migrations (or non-migrants) included in Model A are truncated from our sample for Model B because we only get the first migration characteristics from those who migrated at least once (or only migrants).

The primary explanatory variable in both regression models is ethnicity. We then controlled for individual, origin location, and—only for the all-migrant sample used in Model B—first migration characteristics. The details on how we determined ethnicity from the data can be found in Appendix (Online Resource, Table A.4).

We further categorised the ethnicity based on the number of respondents and geographical proximity. The Sundanese, Bantenese, and Cirebonese were grouped because they used to be in West Java province, which was split after the 1998 reformation. The western part, the home of the Bantenese, is now the province of Banten. The eastern part with the Sundanese majority is still called West Java province, where the Cirebonese also come from. The Buginese and Makassarese were grouped because they are from the province of South Sulawesi. The Sasak, Bima, and Dompu were grouped as they are from West Nusa Tenggara province. We treat ‘others’ as a residual category, which is not further analysed. The final categories of ethnicity are reported in Subsection 4.1, Table 1.^13^

The individual characteristics are birth cohort, gender, education, and socioeconomic status measured by parents’ years of schooling. We account for the geographical aspects by including: (1) the distance of the birth province from Jakarta; (2) the region at age 12, whether in Java or outside Java; and (3) the urbanity of the residential area at age 12, based on the respondent’s assessment as to whether the area constituted a village, small town, or big city to differentiate rural–urban areas at the start of the trajectories.

The first migration characteristics included in Model B are the age of the first migration, the reason for the first migration, the length of stay after the first migration. We interacted parents’ years of schooling with the age of the first migration because the effect of parents’ years of schooling may differ by the age of the first migration as parents with better economic status tend to keep their children at home longer (Whittington & Peters, 1996). The reason for the first migration is divided into work (respondent's work), education (including training), marriage, family (to be closer to family, move with family, death, divorce, sickness), and others (natural disaster, transmigration, housing, independence).

For other timing variables, we use broad periods to avoid multicollinearities with cohort and age. The length of stay at the destination after the first migration is categorised as up to two years or longer. The year of the first migration is included to control for the changes that occurred over time. The number of geographical units might have changed as the number of villages has increased after the 1979 law (Kato, 1989). Thus, those who cross administrative borders recently may have a higher number of migrations than in the past. Profound political and economic changes also occurred in Indonesia in the late 1990s. Indonesia was hit by the financial crisis in the late 1990s, experienced the 1998 political reformation, and started the regional autonomy and fiscal decentralisation in 1999 (Firman, 2004). Therefore, we divide the year of the first migration into before 1980, 1980–1995, and after 1995.

Finally, we included other technical control variables. Only for Model B, a control variable ever migrated abroad within the trajectory was added because time spent abroad implies less exposure to the circumstances in the country and less time in observation and at risk to conduct internal migration. The second variable is the age at the end of the trajectory, divided into four groups: 36–45, 46–55, 56–65, 66 or older, to control for right-censoring. Third, we included the year of entry to the IFLS, controlling for respondents entering the IFLS at different waves.

We estimated seven models for Model A and 11 models for Model B, adding the different controls step-by-step. Model 1 in each regression table highlights the effects of ethnic groups on migration using only birth cohort, accounting for differing shared experiences, and the aforementioned technical control variables.

## Results

### Descriptive Results

The descriptive statistics of the respondents are reported in Table 1 and 2. The highest percentage of ever-migrating respondents is for the Malay and South Sumatra, while the lowest is for the Madurese. The highest mean number of migrations is 2.75 for the Minangkabau, while the lowest is 1.99 for the Betawi (Table 1). Most of the results regarding individual, origin location, and the first migration characteristics seem to be in line with the expectations.

**Table 1:** The percentage of ever-migrating respondents since age 12 (migrants) and the summary statistics of the number of migrations, by variable. *Source:* The 1993, 1997, 2000, 2007, 2014 IFLS, calculated using the respondents with migration history since age 12 who reached at least 36 years

		All respondents		Migrants				
Variables		Migrants	Obs	Number of migrations				Obs
		(%)	(%)	Mean	SD	Min	Max	(%)
*Individual characteristics*								
Ethnicity								
	Batak	67.0	4.0	2.60	1.77	1	11	4.7
	Minangkabau	64.8	5.3	2.75	1.63	1	11	6.0
	Malay & South Sumatra	68.6	4.2	2.51	1.61	1	10	5.0
	Bantenese, Sundanese, & Cirebonese	60.8	13.8	2.28	1.54	1	12	14.7
	Betawi	48.5	3.7	1.99	1.19	1	8	3.1
	Javanese	58.1	44.3	2.38	1.62	1	20	45.2
	Madurese	36.6	3.1	2.05	1.48	1	9	2.0
	Banjarese	62.8	3.1	2.61	1.84	1	12	3.5
	Buginese & Makassarese	51.0	5.2	2.29	1.64	1	16	4.6
	Balinese	43.7	4.6	2.27	1.43	1	9	3.5
	Sasak, Bima & Dompu	44.5	5.3	2.25	1.67	1	12	4.1
	Others	57.4	3.5	2.45	1.62	1	12	3.5
Birth cohort								
	< 1955	49.1	34.7	2.24	1.61	1	16	29.9
	1955–1969	57.9	36.7	2.41	1.64	1	13	37.4
	≥ 1970	65.4	28.5	2.48	1.58	1	20	32.8
Gender								
	Male	60.2	49.2	2.59	1.75	1	20	52.0
	Female	53.9	50.8	2.16	1.42	1	13	48.0
Highest level of education								
	< Primary	43.1	37.7	1.89	1.23	1	12	28.5
	Primary	54.4	23.9	2.22	1.51	1	20	22.8
	Junior	65.1	11.6	2.52	1.66	1	11	13.3
	≥ Senior	75.3	26.8	2.84	1.79	1	16	35.4
Parent's years of schooling								
	< 6 years (< Primary)	49.4	59.5	2.15	1.47	1	20	51.5
	6 years (Primary)	64.2	26.3	2.49	1.64	1	12	29.6
	> 6 years (≥ Junior)	75.7	14.2	2.84	1.82	1	13	18.9
*Location of origin characteristics*								
Distance of the birth province from Jakarta								
	< 250 kms	57.6	23.5	2.24	1.50	1	12	23.7
	250–700 kms	57.4	44.3	2.41	1.63	1	20	44.6
	> 700 kms	56.0	32.3	2.46	1.66	1	16	31.7
Region at age 12								
	Java	56.2	60.2	2.33	1.58	1	20	59.4
	Outside Java	58.2	39.8	2.46	1.65	1	16	40.6
Area at age 12								
	Village	52.9	72.1	2.33	1.58	1	20	67.0
	Small town	65.2	20.0	2.51	1.66	1	12	22.9
	Big city	73.5	7.9	2.47	1.70	1	13	10.2
*First migration characteristics*								
Age group of the first migration (in years)								
	12–17	-	-	3.01	1.89	1	16	28.1
	18–24	-	-	2.52	1.62	1	20	36.5
	≥ 25	-	-	1.74	1.04	1	10	35.4
Main reason for the first migration								
	Work	-	-	2.82	1.63	1	12	25.2
	Education	-	-	3.80	1.87	1	16	10.0
	Marriage	-	-	1.73	1.09	1	10	27.1
	Family	-	-	2.36	1.61	1	20	24.2
	Other	-	-	1.88	1.32	1	12	13.5
Length of stay after the first migration								
	≤ 2 years	-	-	3.29	1.8	1	20	20.0
	> 2 years	-	-	2.16	1.5	1	16	80.0
*Control variables for exposure and timing*								
Ever migrated abroad								
	Yes	-	-	2.10	1.51	1	9	1.6
	No	-	-	2.39	1.61	1	20	98.4
Year of the first migration								
	< 1980	-	-	2.56	1.78	1	16	32.4
	1980–1995	-	-					
	> 1995	-	-	2.58	1.66	1	20	41.8
Age group at the end of observation (in years)								
	36–45	64.6	36.9	2.49	1.59	1	20	41.8
	46–55	56.8	26.5	2.37	1.64	1	13	26.4
	56–65	53.4	19.1	2.32	1.66	1	16	17.9
	≥ 66	45.3	17.5	2.17	1.56	1	12	13.9
Year of entry to IFLS								
	1993	53.2	75.3	2.30	1.56	1	16	70.3
	1997	63.7	6.2	2.45	1.67	1	20	6.9
	2000	69.7	8.0	2.73	1.77	1	12	9.7
	2007	73.0	7.2	2.59	1.66	1	12	9.2
	2014	65.2	3.4	2.52	1.72	1	12	3.9
Total		57.0	100.0	2.38	1.61	1	20	100.0
Observations		21,337						12,161

*SD* standard deviation. The Pearson *χ*^2^ tests of independence for the distribution of all respondents by migrants and non-migrants showed that all variables were significantly related to migration at the five-per cent level, except for the distance of the birth province from Jakarta

**Table 2:** Summary statistics of continuous variables. *Source:* The 1993, 1997, 2000, 2007, 2014 IFLS, calculated using the respondents with migration history since age 12 who reached at least 36 years

Variables		Mean	SD	Min	Max
*All respondents*					
	Parent's years of schooling	3.17	4.23	0	16
	Distance of the birth province from Jakarta (in kms)	622.03	435.01	0	3246.62
*Migrants*					
	Parent's years of schooling	3.90	4.52	0	16
	Age of the first migration	23.83	10.16	12	94
	Distance of the birth province from Jakarta (in kms)	612.88	434.09	0	3246.62

*SD* = standard deviation

It is necessary to point out that controlling for education and parents' education is particularly important. As previously mentioned, Western education impacted each ethnic group differently. Some ethnic groups embraced it more (e.g. the Minangkabau (Tomagola, 1982); the Batak (Rodenburg, 1997)) than the others in the past (e.g. the Betawi (Castles, 1967); the Madurese (Husson, 1997)). It shaped the current levels of education of ethnic groups (Jones et al., 2016), for some quite strikingly, that may affect their contemporary migration patterns. The different levels of education among the ethnic groups can also be observed in the IFLS data (See Appendix, Online Resource, Table A.5.) (Table 3).

### Regression Results

#### Ethnicity and Migration

The regression results of ever migrating, Model A can be seen in Tables 3, 4. Model A1, which contains our ethnicity classification along with controls for exposure and timing, shows that the people belonging to the Batak, Minangkabau, Malay and South Sumatra, Bantenese, Sundanese and Cirebonese, and Banjarese are more likely to ever migrate than those belonging to the Javanese, while the reverse is true for the Betawi, Madurese, Buginese and Makassarese, Balinese, Sasak, Bima and Dompu. However, in the last model (A7), which includes all controls, the difference between the Batak, Minangkabau, Malay and South Sumatra, Bantenese, Sundanese and Cirebonese, and Banjarese with the Javanese becomes not significant or even negative for the case of the Minangkabau. The Betawi, the Madurese, the Buginese and Makassarese, the Balinese, the Sasak, Bima and Dompu are consistently less likely to ever migrate for Model A1-A7 than the Javanese. For the Batak, Minangkabau, Malay and South Sumatra, and Banjarese, adding original location variables changes the significance and even the direction of the effect. Thus, the higher likelihood of migrating of these ethnic groups in Model A1-A4 seems to be partly explained by the distance of their birth province to Jakarta, their region and their type of area at age 12. These results suggest that the geographical location of the ethnic groups shapes their ever-migrating tendencies.

**Table 3:** Model A: Logistic regression odds ratios (OR) and standard errors (SE) of ever migrating since age 12. *Source:* The 1993, 1997, 2000, 2007, 2014 IFLS, calculated using the respondents with migration history since age 12 who reached at least 36 years

Variables		Model A1		Model A2		Model A3		Model A4		Model A5		Model A6		Model A7	
		OR	SE	OR	SE	OR	SE	OR	SE	OR	SE	OR	SE	OR	SE
*Ethnicity (Ref: Javanese)*															
	Batak	1.47***	(0.13)	1.48***	(0.13)	1.25**	(0.11)	1.23**	(0.11)	1.07	(0.11)	0.88	(0.10)	0.87	(0.10)
	Minangkabau	1.32***	(0.11)	1.33***	(0.11)	1.18**	(0.09)	1.17**	(0.09)	1.08	(0.09)	0.83*	(0.08)	0.82**	(0.08)
	Malay & South Sumatra	1.52***	(0.13)	1.52***	(0.13)	1.49***	(0.13)	1.45***	(0.12)	1.46***	(0.13)	1.05	(0.11)	1.07	(0.11)
	Bantenese, Sundanese, & Cirebonese	1.12**	(0.06)	1.12**	(0.06)	1.11**	(0.06)	1.09	(0.05)	1.16***	(0.07)	1.10	(0.06)	1.09	(0.06)
	Betawi	0.67***	(0.06)	0.66***	(0.06)	0.63***	(0.06)	0.63***	(0.06)	0.69***	(0.07)	0.65***	(0.07)	0.56***	(0.06)
	Madurese	0.42***	(0.04)	0.42***	(0.04)	0.51***	(0.05)	0.52***	(0.05)	0.51***	(0.05)	0.54***	(0.05)	0.52***	(0.05)
	Banjarese	1.21*	(0.12)	1.21*	(0.12)	1.29**	(0.13)	1.29**	(0.13)	1.18	(0.13)	0.92	(0.11)	0.89	(0.11)
	Buginese & Makassarese	0.74***	(0.06)	0.74***	(0.06)	0.80***	(0.06)	0.83**	(0.07)	0.70***	(0.07)	0.59***	(0.06)	0.57***	(0.06)
	Balinese	0.56***	(0.05)	0.56***	(0.05)	0.54***	(0.04)	0.56***	(0.05)	0.51***	(0.05)	0.40***	(0.04)	0.40***	(0.04)
	Sasak, Bima, & Dompu	0.56***	(0.05)	0.56***	(0.05)	0.62***	(0.05)	0.64***	(0.05)	0.56***	(0.06)	0.46***	(0.05)	0.46***	(0.05)
	Other	1.02	(0.09)	1.02	(0.09)	0.95	(0.09)	0.94	(0.09)	0.86	(0.08)	0.71***	(0.07)	0.66***	(0.07)
Birth cohort (Ref: < 1955)															
	1955–1969	1.10*	(0.06)	1.12**	(0.06)	1.06	(0.06)	1.04	(0.06)	1.04	(0.06)	1.03	(0.05)	1.04	(0.06)
	≥ 1970	1.21**	(0.09)	1.25***	(0.10)	1.05	(0.08)	1.03	(0.08)	1.03	(0.08)	1.00	(0.08)	1.05	(0.08)
Gender: Male				1.28***	(0.03)	1.14***	(0.03)	1.16***	(0.03)	1.16***	(0.03)	1.16***	(0.03)	1.17***	(0.03)
*Level of education (Ref: < Primary)*															
	Primary					1.38***	(0.05)	1.31***	(0.05)	1.31***	(0.05)	1.32***	(0.05)	1.31***	(0.05)
	Junior					2.08***	(0.11)	1.89***	(0.10)	1.88***	(0.10)	1.89***	(0.10)	1.82***	(0.10)
	≥ Senior					3.34***	(0.15)	2.75***	(0.13)	2.75***	(0.14)	2.79***	(0.14)	2.60***	(0.13)
Parent's years of schooling (YoS)								1.04***	(0.00)	1.04***	(0.00)	1.04***	(0.00)	1.03***	(0.00)
Distance of the birth province from Jakarta (in 000 kms)										1.19**	(0.09)	0.99	(0.08)	1.03	(0.09)
Region at age 12: Outside Java												1.47***	(0.11)	1.46***	(0.11)
*Area at age 12 (Ref: Big city)*															
	Village													0.57***	(0.04)
	*Small town*													0.74***	(0.05)
*Age group at the end of observation (Ref: 36–45)*															
	46–55	0.87**	(0.05)	0.88**	(0.05)	0.97	(0.06)	0.97	(0.06)	0.97	(0.06)	0.97	(0.06)	0.99	(0.06)
	56–65	0.83***	(0.06)	0.85**	(0.06)	1.05	(0.08)	1.06	(0.08)	1.06	(0.08)	1.05	(0.08)	1.08	(0.08)
	≥ 66	0.63***	(0.05)	0.64***	(0.05)	0.88	(0.07)	0.89	(0.07)	0.89	(0.07)	0.89	(0.07)	0.92	(0.08)
*Year of entry to IFLS (Ref: 1993)*															
	1997	1.38***	(0.09)	1.38***	(0.09)	1.37***	(0.09)	1.37***	(0.09)	1.37***	(0.09)	1.37***	(0.09)	1.40***	(0.09)
	2000	1.74***	(0.10)	1.72***	(0.10)	1.68***	(0.10)	1.67***	(0.10)	1.67***	(0.10)	1.67***	(0.10)	1.69***	(0.10)
	2007	1.96***	(0.12)	1.88***	(0.12)	1.76***	(0.11)	1.74***	(0.11)	1.73***	(0.11)	1.73***	(0.11)	1.75***	(0.11)
	2014	1.38***	(0.12)	1.33***	(0.11)	1.28***	(0.11)	1.26***	(0.11)	1.26***	(0.11)	1.27***	(0.11)	1.25***	(0.11)
Constant		1.33***	(0.10)	1.15*	(0.09)	0.73***	(0.06)	0.70***	(0.06)	0.64***	(0.06)	0.68***	(0.06)	1.09	(0.12)
Wald *χ*^2^ (degree of freedom)		934.54 (22)		1007.45 (23)		1603.30 (26)		1635.10 (27)		1639.03 (28)		1664.02 (29)		1704.33 (31)	
Pseudo-*R*^2^		0.0371		0.0396		0.0710		0.0741		0.0744		0.0755		0.0792	
Observations		21,337		21,337		21,337		21,337		21,337		21,337		21,337	

^*^ *p* < 0.10, ** *p* < 0.05, *** *p* < 0.01

The VIF values from the multicollinearity tests are all below 10

**Table 4:** Model B: Truncated negative binomial regression incidence-rate ratio of the number of migrations (standard errors in parentheses). *Source:* The 1993, 1997, 2000, 2007, 2014 IFLS, calculated using the respondents with migration history since age 12 who reached at least 36 years

Variables	B1	B2	B3	B4	B5	B6	B7	B8	B9	B10	B11
*Ethnicity (Ref: Javanese)*											
Batak	1.12**	1.12***	1.05	1.05	1.04	1.03	1.001	1.02	1.01	1.001	1.002
	(0.05)	(0.05)	(0.04)	(0.04)	(0.04)	(0.04)	(0.04)	(0.04)	(0.05)	(0.05)	(0.05)
Minangkabau	1.25***	1.25***	1.17***	1.17***	1.16***	1.16***	1.12***	1.11***	1.11***	1.09**	1.08**
	(0.04)	(0.04)	(0.04)	(0.04)	(0.04)	(0.04)	(0.03)	(0.03)	(0.04)	(0.04)	(0.04)
Malay & South Sumatra	1.06	1.06	1.06	1.05	1.05	1.05	1.05	1.06*	1.06*	1.03	1.03
	(0.04)	(0.04)	(0.04)	(0.04)	(0.04)	(0.04)	(0.04)	(0.04)	(0.04)	(0.04)	(0.04)
Bantenese, Sundanese, & Cirebonese	0.96	0.96	0.96	0.96	0.96	0.96	0.99	1.001	1.002	0.998	0.998
	(0.03)	(0.03)	(0.03)	(0.03)	(0.03)	(0.03)	(0.03)	(0.02)	(0.03)	(0.03)	(0.03)
Betawi	0.81***	0.81***	0.81***	0.81***	0.85***	0.85***	0.93	0.93	0.93	0.92	0.90*
	(0.05)	(0.05)	(0.05)	(0.05)	(0.05)	(0.05)	(0.05)	(0.05)	(0.05)	(0.05)	(0.05)
Madurese	0.81**	0.80***	0.93	0.94	0.95	0.95	0.96	0.99	0.99	0.997	0.99
	(0.07)	(0.07)	(0.08)	(0.08)	(0.08)	(0.08)	(0.07)	(0.07)	(0.07)	(0.07)	(0.07)
Banjarese	1.16***	1.16***	1.19***	1.19***	1.17***	1.17***	1.18***	1.14***	1.14***	1.12**	1.11**
	(0.07)	(0.07)	(0.06)	(0.06)	(0.06)	(0.06)	(0.06)	(0.05)	(0.06)	(0.06)	(0.06)
Buginese & Makassarese	0.98	0.98	1.01	1.02	1.02	1.02	1.03	1.03	1.02	1.01	1.01
	(0.05)	(0.05)	(0.05)	(0.05)	(0.05)	(0.05)	(0.05)	(0.04)	(0.05)	(0.05)	(0.05)
Balinese	0.89**	0.90**	0.86***	0.88**	0.87***	0.87***	0.84***	0.86***	0.86***	0.84***	0.84***
	(0.05)	(0.05)	(0.04)	(0.04)	(0.04)	(0.04)	(0.04)	(0.04)	(0.04)	(0.04)	(0.04)
Sasak, Bima, & Dompu	0.93	0.94	0.98	0.99	0.97	0.97	0.98	1.02	1.02	1.002	1.01
	(0.06)	(0.06)	(0.05)	(0.06)	(0.05)	(0.05)	(0.05)	(0.05)	(0.06)	(0.06)	(0.06)
Other	1.06	1.05	1.01	1.00	1.04	1.04	1.02	1.02	1.02	1.003	0.99
	(0.05)	(0.05)	(0.05)	(0.05)	(0.05)	(0.05)	(0.05)	(0.05)	(0.05)	(0.05)	(0.05)
*Birth cohort: (Ref: < 1955)*											
1955–1969	1.28***	1.30***	1.26***	1.25***	1.04	1.04	1.04	1.05	1.05	1.05	1.05
	(0.05)	(0.05)	(0.04)	(0.04)	(0.04)	(0.04)	(0.03)	(0.03)	(0.03)	(0.03)	(0.03)
≥ 1970	1.61***	1.68***	1.58***	1.56***	1.05	1.05	1.02	1.03	1.03	1.02	1.03
	(0.08)	(0.08)	(0.07)	(0.07)	(0.05)	(0.05)	(0.05)	(0.04)	(0.04)	(0.05)	(0.05)
Gender: Male	1.32***	1.24***	1.25***	1.27***	1.27***	1.15***	1.14***	1.14***	1.14***	1.14***	
		(0.02)	(0.02)	(0.02)	(0.02)	(0.02)	(0.02)	(0.02)	(0.02)	(0.02)	(0.02)
*Level of education (Ref: < primary)*											
Primary			1.27***	1.25***	1.20***	1.21***	1.16***	1.13***	1.13***	1.13***	1.13***
			(0.04)	(0.04)	(0.03)	(0.03)	(0.03)	(0.03)	(0.03)	(0.03)	(0.03)
Junior			1.52***	1.47***	1.40***	1.41***	1.31***	1.26***	1.26***	1.26***	1.26***
			(0.05)	(0.05)	(0.04)	(0.04)	(0.04)	(0.04)	(0.04)	(0.04)	(0.04)
≥ Senior			1.81***	1.69***	1.60***	1.61***	1.40***	1.38***	1.38***	1.38***	1.36***
			(0.05)	(0.05)	(0.04)	(0.04)	(0.04)	(0.04)	(0.04)	(0.04)	(0.04)
Parent's years of schooling (YoS)			1.01***	1.01***	1.04***	1.02***	1.02***	1.02***	1.02***	1.02***	
				(0.002)	(0.002)	(0.01)	(0.01)	(0.01)	(0.01)	(0.01)	(0.01)
Age at the first migration				0.96***	0.96***	0.97***	0.97***	0.97***	0.97***	0.97***	
					(0.002)	(0.002)	(0.002)	(0.002)	(0.002)	(0.002)	(0.002)
Parent's YoS × Age at the first migration					0.999***	0.9995*	0.999**	0.999**	0.999**	0.999**	
					(0.0003)	(0.0003)	(0.0002)	(0.0002)	(0.0002)	(0.0002)	
*Reason for the first migration (Ref: work)*											
Education							1.03	1.10***	1.10***	1.10***	1.10***
							(0.02)	(0.02)	(0.02)	(0.03)	(0.03)
Marriage							0.52***	0.59***	0.59***	0.59***	0.59***
							(0.01)	(0.02)	(0.02)	(0.02)	(0.02)
Family							0.81***	0.87***	0.87***	0.87***	0.86***
							(0.02)	(0.02)	(0.02)	(0.02)	(0.02)
Other							0.68***	0.74***	0.74***	0.74***	0.73***
							(0.02)	(0.02)	(0.02)	(0.02)	(0.02)
							0.59***	0.59***	0.59***	0.59***	
Length of stay after the first migration: > 2 years								(0.01)	(0.01)	(0.01)	(0.01)
Distance of the birth province from Jakarta								1.01	0.99	0.995	
									(0.03)	(0.04)	(0.04)
Region at age 12: Outside Java									1.03	1.03	
										(0.03)	(0.03)
*Area at age 12 (Ref: Big city)*											
Village											0.92***
											(0.03)
Town											0.97
											(0.03)
Ever migrated abroad	0.73***	0.71***	0.72***	0.72***	0.71***	0.71***	0.64***	0.61***	0.61***	0.61***	0.61***
	(0.06)	(0.06)	(0.06)	(0.06)	(0.06)	(0.06)	(0.05)	(0.05)	(0.05)	(0.05)	(0.05)
*Year of first migration (Ref: < 1980)*											
1980–1995	0.66***	0.67***	0.68***	0.68***	1.01	1.01	0.997	0.99	0.99	0.99	0.99
	(0.02)	(0.02)	(0.02)	(0.02)	(0.03)	(0.03)	(0.03)	(0.03)	(0.03)	(0.03)	(0.03)
> 1995	0.35***	0.35***	0.36***	0.36***	0.81***	0.82***	0.86***	0.81***	0.81***	0.81***	0.81***
	(0.01)	(0.01)	(0.01)	(0.01)	(0.04)	(0.04)	(0.04)	(0.04)	(0.04)	(0.04)	(0.04)
*Age at the end of observation (Ref: 36–45)*											
46–55	0.92***	0.93**	0.99	0.99	1.04	1.04	1.03	1.04	1.04	1.04	1.04
	(0.03)	(0.03)	(0.03)	(0.03)	(0.03)	(0.03)	(0.03)	(0.03)	(0.03)	(0.03)	(0.03)
56–65	0.83***	0.85***	0.94	0.95	1.12***	1.12***	1.12***	1.13***	1.13***	1.13***	1.14***
	(0.04)	(0.04)	(0.04)	(0.04)	(0.05)	(0.05)	(0.04)	(0.04)	(0.04)	(0.04)	(0.04)
≥ 66	0.76***	0.78***	0.93	0.94	1.19***	1.19***	1.17***	1.17***	1.17***	1.17***	1.18***
	(0.04)	(0.04)	(0.05)	(0.05)	(0.06)	(0.06)	(0.06)	(0.05)	(0.05)	(0.05)	(0.05)
*Year of entry to IFLS (Ref: 1993)*											
1997	1.04	1.04	1.04	1.04	1.06	1.05	1.05	1.04	1.04	1.04	1.05
	(0.04)	(0.04)	(0.04)	(0.04)	(0.04)	(0.04)	(0.03)	(0.03)	(0.03)	(0.03)	(0.03)
2000	1.23***	1.21***	1.19***	1.19***	1.17***	1.17***	1.14***	1.15***	1.15***	1.15***	1.15***
	(0.04)	(0.04)	(0.03)	(0.03)	(0.03)	(0.03)	(0.03)	(0.03)	(0.03)	(0.03)	(0.03)
2007	1.21***	1.15***	1.11***	1.11***	1.09***	1.08***	1.04*	1.07***	1.07***	1.07***	1.07***
	(0.04)	(0.03)	(0.03)	(0.03)	(0.03)	(0.03)	(0.03)	(0.03)	(0.03)	(0.03)	(0.03)
2014	1.17***	1.12**	1.10**	1.10**	1.09**	1.09**	1.06	1.09**	1.09**	1.09**	1.09**
	(0.05)	(0.05)	(0.05)	(0.05)	(0.04)	(0.04)	(0.04)	(0.04)	(0.04)	(0.04)	(0.04)
Constant	2.25***	1.91***	1.38***	1.35***	2.72***	2.50***	3.34***	4.77***	4.76***	4.78***	5.11***
	(0.11)	(0.09)	(0.07)	(0.06)	(0.15)	(0.15)	(0.19)	(0.26)	(0.27)	(0.27)	(0.32)
lnalpha	0.23***	0.21***	0.15***	0.15***	0.10***	0.10***	0.06***	0.02***	0.02***	0.02***	0.02***
	(0.02)	(0.02)	(0.01)	(0.01)	(0.01)	(0.01)	(0.01)	(0.01)	(0.01)	(0.01)	(0.01)
Wald *χ*^2^	1530.8	2066.8	2131.0	2266.6	2383.3	3404.5	4333.8	4338.6	4339.8	4344.0	4395.6
(degree of freedom)	(23)	(26)	(27)	(28)	(29)	(33)	(34)	(35)	(36)	(38)	(39)
Pseudo-*R*^2^	0.039	0.056	0.057	0.074	0.075	0.095	0.120	0.120	0.120	0.120	0.122

* *p* < 0.10, ** *p* < 0.05, *** *p* < 0.01

The VIF values from the multicollinearity tests are all below 10

In Table 4 (Model B), we report our analysis of the number of migrations across the lifespan for those who moved. Only controlling for the timing and exposure variables (Model B1), we find that the Batak, Minangkabau, and Banjarese have a higher expected number of migrations than the Javanese. For example, the predicted number of migrations is 25 per cent [(1.25–1) × 100] higher for the Minangkabau. Conversely, the Sundanese, Bantenese and Cirebonese, the Betawi, the Madurese, and the Balinese have lower migration counts.

In Model B3, after introducing the education variable, the higher expected number of migrations of the Batak and the lower expected number of migrations of the Madurese become statistically not significant. Thus, for some ethnic groups, the differences reported in Model B1 may be attributed to the difference in education level. It is also clear that this is not a universal pattern: the Minangkabau, for example, exhibit an education level similar to the Batak (See Appendix, Online Resource, Table A.5.), but controlling for education did not substantially alter the finding that the Minangkabau are relatively more mobile.

In Model B7-B9, we added the first migration and some origin location characteristics. For the Betawi, the lower expected number of migrations, found in Models B1-B6, become not significantly different from the Javanese when the reason for the first migration, the length of stay after the first migration, and the distance of the birth province to Jakarta are included.

After including all other variables in Model B11, we find that the Minangkabau and the Banjarese exhibit a higher expected number of moves than the Javanese.^14^ The lower expected number of migrations of the Betawi, which is not significantly different from the Javanese in Model B7-B9, becomes significant again at the 10 per cent level after including whether they resided in the village, small town, or big city. We also find consistently negative effects of belonging to the Balinese on the number of moves relative to the Javanese.

Thus, after controlling all characteristics, the ethnic groups that have a lower likelihood of ever migrating do not always have a lower expected number of migrations than the Javanese and vice versa. The Balinese and the Betawi have a lower likelihood of ever migrating and also a lower expected number of migrations than the Javanese. The Minangkabau even have a combination of a lower likelihood of ever migrating with a higher number of migrations relative to the Javanese. The ethnic groups that are repeatedly mentioned as highly mobile in various studies—the Batak, Minangkabau, Banjarese, and Buginese (together with the Makassarese) (Lineton, 1975; Naim, 1973; Tirtosudarmo, 2009)—do not always have a higher tendency to ever migrate nor higher expected number of migrations, in comparison with the Javanese. In combination, the results of Models A and B show that the differences between the ethnic groups in the likelihood of ever migrating are smaller for the number of migrations.

#### Other Characteristics and Migration

Some of the findings regarding individual characteristics confirm our expectations. Males are consistently more likely to ever migrate than females. Being male also increases the expected number of migrations by 14 per cent [(1.14–1) × 100] (Model A12), even after controlling for the reason of the first migration. This result suggests that this gender gap is not fully explained by the fact that males have a higher tendency to move for work than females (Pardede et al., 2020). As females are more likely to move to more developed areas than males (Wajdi et al., 2017), females may move relatively directly to the areas with better economic opportunities instead of moving repeatedly.

Education is positively related to ever migrating and the number of migrations. Parent’s years of schooling has a nonlinear effect on the number of migrations through the age of the first migration: the effect of age at first migration on migration count becomes less negative at higher levels of parental education.

In Model A7, we do not find evidence for differences in the likelihood of ever migrating between birth cohorts. Inevitably, we have relatively short histories available for the more recent birth cohorts, which favours the identification of young and quick migrants, making the cohort seem relatively mobile.

We do not find evidence that the distance from Jakarta affected migration. However, living outside Java at age 12 positively affects ever migrating as expected, while its effects on the number of migrations are not statistically significant. Also, as expected, living in a village or small town at age 12 reduces the likelihood of ever migrating, which is in line with earlier research (Pardede et al., 2020). On the contrary, starting from the village is related to a lower expected number of migrations than starting from the big city.

In line with our expectations, we find that motives for first migration other than work or education lower the expected number of migrations (Model B11). Migrants who stay at the destination of the first migration longer than two years have a lower expected number of migrations. Lastly, we find a negative effect of ever moved abroad. As expected, moving abroad reduces exposure to domestic moves.

## Conclusion and Discussion

Controlling for a rich set of individual characteristics, exposure, and time, we find evidence that ethnicity is related to migration for the case of Indonesia. The Minangkabau, Betawi, Madurese, Buginese and Makassarese, Balinese, Sasak, Bima and Dompu are less likely to ever migrate than the Javanese. Conversely, belonging to the Minangkabau or the Banjarese is positively related to the number of migrations, while the opposite is true for the Balinese and the Betawi. With these findings, we show that there are differences between the ethnic groups in the likelihood of ever migrating. However, we find less differences between the ethnic groups regarding the number of migrations. Furthermore, we find that a lower likelihood of ever migrating does not always correspond with a lower number of migrations.

Our results reflect the four principles identified in this paper as potential drivers of the ethnic differences in migration. Firstly, our results suggest that the effect of ethnicity on ever migrating can be attributed to the effect of the home region being in the centre or periphery and on the impacts of urban centres created since the colonial era. We find that the ethnic groups commonly mentioned as highly mobile, such as the Minangkabau and the Buginese, have a similar likelihood of ever migrating to the groups commonly considered to have lower intensity of migration, such as the Balinese and the Sasak. We find that all these ethnic groups exhibit a lower likelihood of ever migrating than the Javanese, who are from the central regions. The Betawi, originally from Jakarta, have a lower likelihood of ever migrating than the Javanese and exhibit a lower number of migrations. The results are not surprising for the Betawi because they tend to move to the surrounding provinces of Jakarta (Jones et al., 2016). The need to move again is perhaps lower because they continue to live near Jakarta.

Our results also suggest that ethnicity may play a role as a cultural marker of migration behaviour, potentially driven by the average migration skills possessed by an ethnic group. Regarding the culturally determined differentials of migration by ethnicity in Indonesia, Titus said, 'not so much the cultural differences that are decisive, but much more the specific role assigned to the various regions and peoples in the process of peripheral capitalistic development' (1978, p. 202). Still, after including geographical aspects on top of controlling for various personal characteristics, we find that ethnicity affects migration across the lifespan.

Our results also suggest relevant roles of ethnically segmented migration networks and labour markets on migration for some ethnic groups, aligned with international literature regarding social capital (Reynolds, 2010). We indeed find that the ethnic groups traditionally more engaged in trade (Chadwick, 1991; Hawkins, 2000) have a higher number of migrations, such as the Minangkabau and the Banjarese. The lower expected number of migrations of the Balinese may be related to their tendency to move to the locations where previous migrants have established their social system (Roth, 2011). They may not need to move again because living in a destination with an established Balinese social system may inhibit more migrations. Aside from intraethnic social capital, however, one interesting line of enquiry would be whether interethnic social capital also plays roles in migration, especially as interethnic relation is also important in determining migrants’ socioeconomic status (Riedel, 2015).

Regarding the data limitation, we analysed mainly the largest ethnic groups as we focused on the ethnic groups residing in the IFLS provinces, as previously discussed. The smaller ethnic groups were also captured; among those are the migrants and their descendants from the non-IFLS provinces. As these groups are small in size, our results as a whole were not affected that much. Still, it would be worth investigating other ethnic groups' migration behaviour, particularly in the middle and eastern parts of Indonesia.

We also note that cultural identities, such as ethnicity, are fluid and 'not fixed in space and time' (Hawkins, 2000, p. 25). Thus, the ethnicity defined in the data should be assessed further, mainly on how it affects migration. For example, the members of other ethnic groups in Kalimantan identified themselves as Banjarese when they converted to Islam (Hawkins, 2000) might have influenced the Banjarese migration rates.

Another issue that needs to be mentioned is that we included various time elements in the analysis. Hence, we have partly isolated the changes across the age, period, and cohort, such as the stages of development and changes through the trajectories. Still, the members of specific ethnic groups may have experienced changes in migration behaviour over time as the migration assessed is the lifespan migration since age 12 and also covers different cohorts. Therefore, further analysis is needed to address the issue of changes in migration behaviour. Following De Haas (2010), migration aspirations and capabilities may change and need further assessment..

This paper also adds to earlier studies on migration as a one-time event phenomenon by distinguishing between the likelihood of ever migrating and the number of migrations across the lifespan and how the effect of ethnicity on migration differs for these two measures. Doing so provides a point of departure to understand the phenomena of repeat migrations and migration trajectories in general, in multi-ethnic countries, in developed countries with issues regarding ethnicity and international migrations, and in Indonesia in particular.

## Supplementary Information

Below is the link to the electronic supplementary material.

Supplementary file1 (PDF 341 kb)

## Data Availability

The datasets generated and/or analysed during the current study are available in the RAND repository, https://www.rand.org/well-being/social-and-behavioral-policy/data/FLS/IFLS.html.

## Footnotes

1 In the case of Indonesia, ethnic diversity is far greater and much more complex than what occurs elsewhere. For example, there are around 2,500 languages spoken, recorded by the 2010 Census (BPS, 2011). While about 90 per cent of the population aged five and older can speak the national language, Indonesian, only 20 per cent use it as their daily spoken language at home (Ananta et al., 2015; BPS 2011). Furthermore, although the country is predominantly Muslim, most people of some ethnic groups in their home regions tend to have a particular other religion. Thus, to a great extent, ethnicity is largely overlapped with religion (Van Klinken, 2003).

2 We follow the English names of the ethnic groups used by Ananta et al. (2015), while some studies use slightly different English names for some. The islands of origin of the ethnic groups are taken from the classification by BPS (2011).

3 Following BPS (2011), South Sumatra here does not refer to a location, but an ethnic group category, consisting of various ethnic groups from South Sumatra province, such as Palembang and Komering.

4 The intensity of migration was measured by lifetime migration, which is the percentage of people living in other than their district of birth (Nitisastro, 2006), the same level as *kewedanan* (Hugo, 1978).

5 The Enumeration Areas in the IFLS were randomly selected from the nationally representative sample frame of the 1993 National Socioeconomic Survey designed by BPS based on the 1990 census (Frankenberg & Karoly, 1995). An enumeration area in the 1990 census is defined as a portion of a village administrative area with clear permanent physical natural or man-made boundaries, covering on average 200–300 households (BPS, 1992).

6 The migration definition in the IFLS involves staying for at least six months in the destination, which can be categorised as permanent migration. In the text, we use the terms moving and migration interchangeably.

7 Auwalin (2020) calculated the migration rates based on five-year migration rates at the provincial level (change of provincial residence within five years prior to the census). He displays only the rates of the ten largest ethnic groups. We also calculated the percentage of the ethnic groups living outside their home province in Appendix, Online Resource, Table A.1, using the 2010 Census publication (BPS, 2011) alongside the 1930 results (Naim, 1973). We find that the ranks of the ethnic groups by the proportions of migrant stocks are somewhat similar to the 1930 Census results.

8 We acknowledge that although these censuses depict somewhat similar patterns, the context of migration (such as norms/attitudes) and migration aspirations and capabilities (De Haas, 2010), might have changed overtime.

9 Migration networks and segmented labour markets may be established in a lower-level unit, such as a village within a particular ethnic group’s home region (Chadwick, 1991; Skeldon, 1990). However, the mechanism of creating networks and segmented labour market could be extended to a particular ethnic group, as a sense of identity may play a role.

10 According to Cunningham (1958, pp. 3–4), Batak ethnic group (*suku*) consists of several major sub-ethnic groups (*sub-suku*), which are: Toba, Pak-pak, Karo, Simelungun, Angkola, and Mandailing. The studies referred here are about Toba-Batak (Bruner, 1972; Cunningham, 1958; Rodenburg, 1997). Because most statistics do not differentiate these sub-groups (such as in Naim (1973) or Hugo (1978)), except later, such as in Jones et al. (2016), we grouped them as Batak.

11 The Betawi is considered from Jakarta, while they were 'formed' by a mix of migrants from the Outer Islands and the foreigners—many were slaves—who lived in Jakarta during the Dutch colonisation period (Castles, 1967).

12 Data and documentation are accessible at https://www.rand.org/well-being/social-and-behavioral-policy/data/FLS/IFLS.html.

13 The IFLS sample was not designed to represent ethnic groups in Indonesia. Nevertheless, for comparison, we show that the proportion of each ethnic group in the pooled IFLS data and Census 2010 are reasonably similar inAppendix, Table A.1.

14 The higher migration count of the Banjarese needs to be assessed further. Hawkins (2000) noted that ethnic groups other than Banjarese in Kalimantan who converted to Islam tended to identify themselves as the Banjarese. This change in self-identification of ethnicity may partly explain Ananta et al. (2015) finding that only 65 per cent of the Banjarese accounted in Census 2010 resided in their province of origin, while about 20 per cent of them lived in the neighbouring provinces.

## References

[CR1] Ananta, A., Arifin, E. N., Hasbullah, M. S., Handayani, N. B., & Pramono, A. (2015). *Demography of Indonesia's ethnicity.* Singapore: Institute of Southeast Asian Studies.

[CR2] Auwalin, I. (2020). Ethnic identity and internal migration decision in Indonesia. *Journal of Ethnic and Migration Studies,**46*(13), 2841–2861. 10.1080/1369183X.2018.1561252

[CR3] Badan Pusat Statistik. (2011). *Kewarganegaraan, suku bangsa, agama, dan bahasa sehari-hari penduduk Indonesia [Citizenships, ethnicity, religion, and daily language of population of Indonesia].* Jakarta: Badan Pusat Statistik. Retrieved from http://sp2010.bps.go.id/files/ebook/kewarganegaraan%20penduduk%20indonesia/index.html

[CR4] Bazzi, S., Gaduh, A., Rothenberg, A. D., & Wong, M. (2019). Unity in diversity? How intergroup contact can foster nation building. *American Economic Review,**109*(11), 3978–4025. 10.1257/aer.20180174

[CR5] Biro Pusat Statistik. (1992). *Population of Indonesia: Results of the 1990 Population Census. Series S, Number 2.* Jakarta: Biro Pusat Statistik.

[CR6] Borjas, G. J. (1992). Ethnic capital and intergenerational mobility. *The Quarterly Journal of Economics,**107*(1), 123–150. 10.2307/2118325

[CR7] Boyle, P., Halfacree, K., & Robinson, V. (1998). *Exploring contemporary migration*. Addison Wesley Longman Ltd.

[CR8] Bruner, E. M. (1972). Batak ethnic associations in three Indonesian cities. *Southwestern Journal of Anthropology,**28*(3), 207–229. 10.1086/soutjanth.28.3.3629220

[CR9] Castles, L. (1967). The ethnic profile of Djakarta. *Indonesia,**3*, 153–204. 10.2307/3350726

[CR10] Chadwick, R. J. (1991). Matrilineal inheritance and migration in a Minangkabau community. *Indonesia, 51*, 47–82. Retrieved from https://hdl.handle.net/1813/53954

[CR11] Cunningham, C. E. (1958). *The postwar migration of the Toba-Bataks to East Sumatra*. Yale University.

[CR12] DaVanzo, J. (1981). Repeat migration, information costs, and location-specific capital. *Population and Environment,**4*(1), 45–73. 10.1007/BF01362575

[CR13] DaVanzo, J. (1983). Repeat migration in the United States: Who moves back and who moves on? *The Review of Economics and Statistics,**65*(4), 552–559. 10.2307/1935923

[CR22] De Haas, H. (2010). Migration transitions: A theoretical and empirical inquiry into the developmental drivers of international migration. *International Migration Institute**Working Paper**24*. Retrieved from https://www.migrationinstitute.org/publications/wp-24-10

[CR14] De Jonge, H., & Nooteboom, G. (2006). Why the Madurese? Ethnic conflicts in West and East Kalimantan compared. *Asian Journal of Social Science,**34*(3), 456–474. 10.1163/156853106778048597

[CR15] Douglass, M. (1997). Structural change and urbanization in Indonesia: From the “old” to the “new” international division of labour. In G. W. Jones & P. M. Visaria (Eds.), *Urbanization in large developing countries: China, Indonesia, Brazil, and India* (pp. 111–141). Oxford University Press.

[CR16] Elder, G. H. (1975). Age differentiation and the life course. *Annual Review of Sociology,**1*(1), 165–190. 10.1146/annurev.so.01.080175.001121

[CR17] Firman, T. (1997). Patterns and trends of urbanisation: A reflection of regional disparity. In G. W. Jones & T. H. Hull (Eds.), *Indonesia assessment: Population and human resources* (pp. 101–117). Research School of Pacific and Asian Studies, Australian National University, Institute of Southeast Asian Studies.

[CR18] Firman, T. (2004). Demographic and spatial patterns of Indonesia’s recent urbanisation. *Population, Space and Place,**10*(6), 421–434. 10.1002/psp.339

[CR19] Frankenberg, E., & Karoly, L. (1995). *The 1993 Indonesian family life survey Overview and field report.* RAND. DRU-1195/1-NICHD/AID.

[CR20] Geertz, C. (1967). Tihingan: A Balinese village. In Koentjaraningrat (Ed.), *Villages in Indonesia.* Cornell University Press.

[CR21] Grant, E. K., & Vanderkamp, J. (1986). Repeat migration and disappointment. *Canadian Journal of Regional Science, 9*(3), 299–322. Retrieved from http://www.cjrs-rcsr.org/archives/9-3/Grant-Vanderkamp.pdf 12157895

[CR23] Hawkins, M. (2000). Becoming Banjar. *The Asia Pacific Journal of Anthropology,**1*(1), 24–36. 10.1080/14442210010001705830

[CR24] Hugo, G. J. (1978). *Population mobility in West Java*. Gadjah Mada Unversity Press and Department of Demography Australian National University.

[CR25] Hugo, G. J. (1988). Population movement in Indonesia since 1971. *Tijdschrift voor Economische en Sociale Geografie,**79*(4), 242–256. 10.1111/j.1467-9663.1988.tb01310.x 12315534 10.1111/j.1467-9663.1988.tb01310.x

[CR26] Hugo, G. J. (2006). Forced migration in Indonesia: Historical perspectives. *Asian and Pacific Migration Journal,**15*(1), 53–92. 10.1177/011719680601500104

[CR27] Husson, L. (1997). Eight centuries of Madurese migration to East Java. *Asian and Pacific Migration Journal,**6*(1), 77–102. 10.1177/011719689700600105 12321185 10.1177/011719689700600105

[CR28] Jones, G. W., Rangkuti, H., Utomo, A., & McDonald, P. (2016). Migration, ethnicity, and the educational gradient in the Jakarta Mega-Urban Region: A spatial analysis. *Bulletin of Indonesian Economic Studies,**52*(1), 55–76. 10.1080/00074918.2015

[CR29] Kato, T. (1989). Different fields, similar locusts: Adat communities and the Village Law of 1979 in Indonesia. *Indonesia,**47*, 89–114. 10.2307/3351077

[CR30] Lineton, J. (1975). Pasompe’ Ugi’: Bugis migrants and wanderers. *Archipel,**10*, 173–201. 10.3406/arch.1975.1248

[CR31] Long, J. S., & Freese, J. (2014). *Regression models for categorical dependent variables Using STATA* (3rd ed.). StataCorp LP.

[CR32] Long, J. S. (1997). *Regression models for categorical and limited dependent variables.* SAGE Publication.

[CR33] Morrison, P. A. (1971). Chronic movers and the future redistribution of population: A longitudinal analysis. *Demography,**8*(2), 171–184. 10.2307/2060607 5163988

[CR34] Muttaqien, A., Sologon, D., and O'Donoghue, C. (2018). Earnings polarization, ethnicity, and regional perspective in Indonesia. *WIDER Working Paper, No. 2018/106*, pp. 1–23. 10.35188/UNU-WIDER/2018/548-0

[CR35] Naim, M. (1973). *Merantau: Minangkabau voluntary migration.* Faculty of Arts and Social Sciences. Singapore: Unversity of Singapore.

[CR36] Nitisastro, W. (2006). *Population trends in Indonesia (1st* (Equinox). Equinox Publishing.

[CR37] Pardede, E. L., McCann, P., & Venhorst, V. A. (2020). Internal migration in Indonesia: New insights from longitudinal data. *Asian Population Studies*, 1–23. 10.1080/17441730.2020.1774139

[CR38] Rangkuti, H. (2016). *Migration out of Central Java: 1971–2010*. PhD Thesis. Canberra, Australian National University. 10.25911/5d7788929a911

[CR39] Reynolds, T. (2010). Transnational family relationships, social networks and return migration among British-Caribbean young people. *Ethnic and Racial Studies,**33*(5), 797–815. 10.1080/01419870903307931

[CR40] Riedel, S. (2015). The interrelation of immigrants’ interethnic ties and socioeconomic status in Germany. An autoregressive panel analysis. *European Journal of Population*, *31*(3), 287–307. 10.1007/s10680-014-9334-9

[CR41] Rodenburg, J. (1997). *In the shadow of migration: Rural women and their households in North Tapanuli, Indonesia*. KITLV Press.

[CR42] Roth, D. (2011). The Subak in diaspora: Balinese farmers and the Subak in South Sulawesi. *Human Ecology,**39*(1), 55–68. 10.1007/s10745-010-9374-7 10.1007/s10745-010-9374-7 PMC3055986 21475721

[CR43] Skeldon, R. (1990). *Population mobility in developing countries: A reinterpretation*. Belhaven Press.

[CR44] Skeldon, R. (1992). On mobility and fertility transitions in East and Southeast Asia. *Asian and Pacific Migration Journal,**1*(2), 220–249. 10.1177/011719689200100203 10.1177/011719689200100203 12343909

[CR45] Strauss, J., Witoelar, F., & Sikoki, B. (2016). *The fifth wave of the Indonesia family life survey (IFLS5): Overview and field report.* RAND. WR-1143/1-NIA/NICHD.

[CR46] Suryadarma, D., Widyanti, W., Suryahadi, A., & Sumarto, S. (2012). From access to income: Regional and ethnic inequality in Indonesia. In A. Booth, C. Manning, & T. K. Wie (Eds.), *Land, livelihood, the economy and the environment in Indonesia: Essays in honour of Joan Hardjono* (pp. 103–123). Yayasan Pustaka Obor Indonesia. Retrieved from http://hdl.handle.net/1885/57284

[CR47] Tirtosudarmo, R. (2009). *Mobility and human development in Indonesia.* United Nations Development Programme. Retrieved from http://hdr.undp.org/en/content/mobility-and-human-development-indonesia

[CR48] Titus, M. J. (1978). Interregional migration in indonesia as a reflection of social and regional inequalities. *Tijdschrift voor Economische en Sociale Geografie,**69*(4), 194–204. 10.1111/j.1467-9663.1978.tb00853.x 12157774 10.1111/j.1467-9663.1978.tb00853.x

[CR49] Tomagola, T. A. (1982).* The differential educational characteristics of West Sumatran and West Javanese migrants in Jakarta: A socio-historical approach*. PhD Thesis. Canberra, Australian National University. 10.25911/5d723e0118299

[CR50] Tumonggor, M. K., Karafet, T. M., Hallmark, B., Lansing, J. S., Sudoyo, H., Hammer, M. F., & Cox, M. P. (2013). The Indonesian archipelago: An ancient genetic highway linking Asia and the Pacific. *Journal of Human Genetics,**58*(3), 165–173. 10.1038/jhg.2012.154 23344321 10.1038/jhg.2012.154

[CR51] Utomo, A. J., & McDonald, P. F. (2021). Internal migration, group size, and ethnic endogamy in Indonesia. *Geographical Research,**59*, 56–77. 10.1111/1745-5871.12433

[CR52] Van Klinken, G. (2003). Ethnicity in Indonesia. In C. Mackerras (Ed.), *Ethnicity in Asia* (pp. 64–87). Routledge.

[CR53] Wajdi, N., Mulder, C., & Adioetomo, S. (2017). Inter-regional migration in Indonesia: A micro approach. *Journal of Population Research,**34*(3), 253–277. 10.1007/s12546-017-9191-6

[CR54] Wajdi, N., van Wissen, L. J., & Mulder, C. H. (2015). Interregional migration flows in Indonesia. *Sojourn: Journal of Social Issues in Southeast Asia, 30*(2), 371–422. 10.1355/sj30-2c

[CR55] Whittington, L. A., & Peters, H. E. (1996). Economic incentives for financial and residential independence. *Demography,**33*(1), 82–97. 10.2307/2061715 8690142

[CR56] Wingens, M., de Valk, H., Windzio, M., & Aybek, C. (2011). The sociological life course approach and research on migration and integration. In M. Wingens, H. de Valk, M. Windzio, & C. Aybek (Eds.), *A life-course perspective on migration and integration* (pp. 1–26). Springer. 10.1007/978-94-007-1545-5

[CR57] Zelinsky, W. (1971). The hypothesis of the mobility transition. *Geographical Review,**61*(2), 219–249. 10.2307/213996

[CR58] Zhu, Y. (2018). Advancing research on internal migration in Asia: The mobility transition hypothesis revisited. *Asian Population Studies,**14*(1), 1–4. 10.1080/17441730.2017.1328862

