# Supplementary material for: Does Ethnicity Affect Ever Migrating and the Number of Migrations? The Case of Indonesia
Source: Eur J Popul. 2024 Jan 30;40(1):6. doi: 10.1007/s10680-023-09694-z (PMC10828312; doi:10.1007/s10680-023-09694-z)
Supplement: Supplementary file 1 — Supplementary file1 (PDF 341 kb) [file 10680_2023_9694_MOESM1_ESM.pdf]

Appendix

Does ethnicity affect ever migrating and the number of migrations? The case of Indonesia

Elda Luciana Pardede[1,2] & Viktor Andreas Venhorst[3]

European Journal of Population (Supplemental Online Resource)

1) Population Research Centre, Faculty of Spatial Sciences, University of Groningen, Groningen, The Netherlands
2) Master's Programme in Population and Labour Economics, Department of Economics, Faculty of Economics and Business, Universitas Indonesia, Depok, Indonesia
3) Department of Economic Geography, Faculty of Spatial Sciences, University of Groningen, The Netherlands.
   Corresponding author: v.a.venhorst@rug.nl

The comparison of ethnic distribution between the IFLS and Census 2010

We present the distribution of the largest ethnic groups in Indonesia by province of origin (home province) based on the Census 2010 in Table A.1, which corresponds with the map of the distribution of the ethnic groups in the IFLS and non-IFLS provinces (Figure 1). The eight largest ethnic groups in 2010 in descending order are the: (1) Javanese, (2) Sundanese, (3) Batak, (4) Madurese, (5) Betawi, (6) Minangkabau, (7) Buginese, and (8) Malay. These ethnic groups are also available in the IFLS data, although there are minor differences in the relative frequencies between IFLS and Census 2010. For example, the proportions of the ethnic groups in the IFLS are somewhat higher for the Batak, Minangkabau, Betawi, Balinese, Sasak, Banjarese, and Buginese, but slightly lower for the Bantenese, Sundanese, and Cirebonese. Some of the ethnic groups originally from outside of the IFLS provinces are not captured in the IFLS

data such as the ethnicities from East Nusa Tenggara and Papua.

From Table A.1 it becomes clear that through the IFLS we captured most of the eight largest ethnic groups, except for about one-third of the Buginese and one-fifth of the Malay people. Following the 2010 Census ethnic distribution, there are 26.75 per cent of population living in the rest of the non-IFLS provinces. Many of the smaller ethnic groups were grouped as ‘others’ and were treated as a residual category.

Several things should be noted regarding the spatial distribution of these ethnic groups. Firstly, in 2010, the members of the ethnic groups might live in their home provinces or elsewhere. Therefore, we calculated the top ten ethnicities by province and island in 2010. The results are presented in Table A.2. The shaded provinces are the IFLS provinces. We can see, for example, that the Minangkabaus are dominant in their home province, West Sumatra. They comprise 87 per cent of the population in that province. The share of the Batak people in their home province North Sumatra is only 45 per cent. We can also see the Batak people make 3.4 per cent of the population in DKI Jakarta in 2010.

We find high proportions of Javanese in North Sumatra and Lampung in Table A.2 that can indicate how past migrations shape the current migration situations. In 2010, the Javanese are the largest ethnic group in the whole of Sumatra, comprising almost one-third of the population of Sumatra. They are the second largest ethnic group in most Sumatra provinces, reaching 64 per cent in Lampung. The impact of colonisation on the migrant stock (if we consider ethnic groups not staying in their home region) can be seen from this data. Lampung, for example, was established by the Dutch as a transmigration site. Furthermore, the move of many Javanese labour migrants during the colonial time has contributed to the Javanese being the second

largest ethnic group in North Sumatra. Of course, the people who currently live in a province could be migrants and also the descendants of migrants.

We can also see that some of the ethnic groups on the island of Sumatra such as Jambi, and in the eastern parts of Indonesia such as East Nusa Tenggara, and some ethnic groups in Sulawesi, Maluku, and Papua are outside the IFLS provinces. But some of them may have resided in the IFLS provinces. We need to note that we have the experience of the members of these ethnic groups who have already moved or the descendants of migrants. We could miss, however the experience of some mobile ethnic groups such as a quarter of the Banjarese, few of the Makassarese, and one-third of Buginese. But we did not lose the majority of other ethnic groups.

Two provinces with high lifetime migration rates, East Kalimantan and West Papua (Rangkuti, 2016; Utomo & McDonald, 2021), are also not in the IFLS. Many Javanese are currently living in these provinces due to transmigration and voluntary migrations in the past. Nevertheless, we were still able to analyse the ethnic groups from non-IFLS provinces residing in the IFLS provinces but with very low percentages such as Maluku people in South Sulawesi, while totally excluding others such as from East Nusa Tenggara and Papua.

Overall, (1) we have reliable information on migration of the larger ethnic groups than the smaller ethnic groups in the IFLS (particularly among those who originated from outside the IFLS provinces); however (2) we might have analysed the experience of migrants or descendants of migrants who have resided in the IFLS provinces who commonly tend to have higher likelihood of migrating than non-migrants; and (3) we could not assess the migration experience of ethnic groups from eastern parts of Indonesia.

Table A.1. The distribution of the largest ethnic groups (%) by the home province and the proportion of each ethnic group outside the IFLS provinces (%), Census 2010 and IFLS

No.	Ethnic group	Distribution		Living outside the	Home province[a)]	
		PC 2010 (%)	IFLS (%)	IFLS provinces in 2010 (%)	Province in 1993	Province in 2010
1	Aceh*	1.7	0.1	93.6	Aceh	Aceh
2	Batak	3.6	4.8	4.7	North Sumatra	North Sumatra
3	Nias	0.4	0.6	1.5	North Sumatra	North Sumatra
4	Malay	2.3	0.8	21.4	North Sumatra	North Sumatra
					Riau	Riau & Riau Islands
					Jambi	Jambi
					Bengkulu	Bengkulu
					South Sumatra	South Sumatra & Bangka Belitung
					West Kalimantan[b)]	West Kalimantan
5	Minangkabau	2.7	5.6	4.6	West Sumatra	West Sumatra
6	Jambi*	0.6	-	95.1	Jambi	Jambi
7	South Sumatra*	2.2	4.4	4.7	South Sumatra	South Sumatra & Bangka Belitung
8	Lampung*	0.6	-	1.8	Lampung	Lampung
9	Other Sumatra	0.9	-	43.3	-	-
10	Betawi	2.9	4.2	0.2	DKI Jakarta	DKI Jakarta
11	Banten*	2.0	0.3	0.1	West Java	Banten
12	Sundanese	15.5	12.8	1.0	West Java	West Java
13	Cirebonese	0.8	0.4	4.9	West Java	West Java
14	Javanese	40.2	40.9	0.1	West Java	West Java
					Central Java	Central Java
					DI Yogyakarta	DI Yogyakarta
15	Madurese	3.0	2.8	5.3	East Java	East Java

Table A.1., continued.

No.	Ethnic group	Distribution		Living outside the	Home province[a)]	
		PC 2010 (%)	IFLS (%)	IFLS provinces in 2010 (%)	Province in 1993	Province in 2010
16	Balinese	1.7	4.2	5.5	East Java	East Java
17	Sasak	1.3	4.0	2.0	Bali	Bali
18	Other West Nusa Tenggara	0.5	2.5	3.2	West Nusa Tenggara	West Nusa Tenggara
19	East Nusa Tenggara*	1.8	-	94.4	West Nusa Tenggara	West Nusa Tenggara
20	Dayak	1.3	0.1	95.1	East Nusa Tenggara	East Nusa Tenggara
					West Kalimantan	West Kalimantan
					Central Kalimantan	Central Kalimantan
					South Kalimantan	South Kalimantan
					East Kalimantan	East Kalimantan & North Kalimantan
21	Banjarese	1.7	3.2	25.0	South Kalimantan	South Kalimantan
22	Other Kalimantan	0.8	0.001	89.9	-	-
25	Minahasa	0.5	0.1	91.6	North Sulawesi	North Sulawesi
26	Gorontalo	0.5	0.001	98.7	North Sulawesi	Gorontalo
23	Makassarese	1.1	1.6	7.2	South Sulawesi	South Sulawesi
24	Buginese	2.7	4.2	33.3	South Sulawesi	South Sulawesi
27	Other Sulawesi	3.2	0.9	64.0	-	-
28	Maluku*	0.9	0.1	92.4	Maluku	Maluku
					Maluku	North Maluku
29	Papua*	1.1	-	95.2	Irian Jaya	West Papua
					Irian Jaya	Papua
30	Chinese	1.2	1.1	17.4	-	-
31	Foreigners	0.1	-	19.1	-	-
32	Other	-	0.6			
	TOTAL	100.0	100.0			

Source: Census 2010 figures were calculated from BPS (2011) and author's calculation using the IFLS 1993, 1997, 2000, 2007, and 2014

Note: Some of the grouping of ethnicity using the Census 2010 is by BPS (2011) based on detailed categorisation. Based on the IFLS answers, only Sasak, Bima, and Dompu were grouped for West Nusa Tenggara province. For the census case, other ethnic groups in the province are also included. *) Including all ethnic groups from that area. For example, Maluku* includes Ambon, Ternate, and Tidore, among many others; a) The home province names are displayed twice, following the 1993 and 2010 classifications; b) There are 19 per cent of the Malay population in West Kalimantan (see Table A.2) and their presence has long been established there. According to Ananta et al. (2015), Dayak Melayu Pontianak and Dayak Melayu Sambas that were grouped as Dayak by BPS (2011) should also be grouped as Malay. Because of these considerations, we assume that these two sub-ethnic groups of Malay belong to Malay and originally from West Kalimantan.

Table A.2. Percentage (%) of the top ten ethnicities by province and island, Census 2010 (Total Population: 236.73 million)

Rank	SUMATRA									
	Aceh (%)		North Sumatra (%)		West Sumatra (%)		Riau[a)] (%)		Jambi (%)	
1	Aceh[b)]	85.4	Batak	44.8	Minangkabau	87.3	Malay	32.5	Jambi[d)]	43.6
2	Javanese	8.9	Javanese	33.4	Batak	4.6	Javanese	28.2	Javanese	29.1
3	Batak	3.3	Nias	7.1	Javanese	4.5	Batak	12.5	Malay	5.4
4	Minangkabau	0.7	Malay	6.0	Other Sumatra[c)]	1.4	Minangkabau	11.7	Minangkabau	5.3
5	Malay	0.5	Chinese	2.6	Malay	0.8	Banjarese	3.3	Batak	3.5
6	Sundanese	0.2	Minangkabau	2.6	Nias	0.4	Chinese	3.2	Banjarese	3.3
7	Chinese	0.2	Aceh[b)]	1.0	Sundanese	0.3	Buginese	2.0	Buginese	3.1
8	Nias	0.2	Banjarese	1.0	Chinese	0.2	Sundanese	1.8	Sundanese	2.6
9	Papua	0.1	Banten[h)]	0.4	Jambi[d)]	0.1	Nias	1.1	South Sumatra[f)]	1.9
10	Banjarese	0.1	Sundanese	0.3	Lampung[e)]	0.1	Other Sumatra[c)]	0.9	Chinese	1.2
	Other	0.3	Other	1.0	Other	0.2	Other	2.9	Other	1.0
	All	100.0		100.0		100.0		100.0		100.0
% of Total Population		1.9		5.5		2.0		3.0		1.3

Rank	SUMATRA, Continued.							
	South Sumatra[g)] (%)		Bengkulu (%)		Lampung (%)		All Sumatra (%)	
1	South Sumatra[f)]	46.6	Other Sumatra[c)]	55.1	Javanese	64.1	Javanese	30.2
2	Javanese	26.5	Javanese	22.6	Lampung[e)]	13.6	Batak	14.5
3	Other Sumatra[c)]	11.4	South Sumatra[f)]	8.4	Sundanese	9.6	Minangkabau	11.5
4	Malay	7.1	Minangkabau	4.2	South Sumatra[f)]	5.4	South Sumatra[f)]	9.6
5	Sundanese	2.4	Sundanese	3.1	Banten[h)]	2.3	Malay	8.0
6	Minangkabau	1.5	Malay	2.8	Balinese	1.4	Aceh[b)]	7.9
7	Batak	0.9	Batak	1.9	Minangkabau	0.9	Other Sumatra[c)]	4.1

Table A.2., continued.

Rank	SUMATRA, Continued.							
	South Sumatra[g]) (%)		Bengkulu (%)		Lampung (%)		All Sumatra (%)	
8	Chinese	0.8	Lampung[e)]	0.4	Batak	0.7	Jambi[d)]	2.7
9	Lampung[e)]	0.6	Balinese	0.3	Chinese	0.5	Sundanese	2.4
10	Buginese	0.5	Buginese	0.2	Other Sumatra[c)]	0.4	Lampung[e)]	2.2
	Other	1.7	Other	1.0	Other	1.2	Other	6.8
	All	100.0		100.0		100.0		100.0
% of Total Population		3.7		0.7		3.2		21.3

Rank	JAVA											
	DKI Jakarta (%)		West Java[i)] (%)		Central Java (%)		DI Yogyakarta (%)		East Java (%)		All Java (%)	
1	Javanese	36.2	Sundanese	62.1	Javanese	97.7	Javanese	96.5	Javanese	80.7	Javanese	55.7
2	Betawi	28.3	Javanese	13.8	Sundanese	1.4	Sundanese	0.7	Madurese	17.5	Sundanese	25.9
3	Sundanese	14.6	Banten[h)]	8.2	Chinese	0.4	Malay	0.5	Chinese	0.7	Betawi	5.0
4	Chinese	6.6	Betawi	7.5	Batak	0.1	Chinese	0.3	Batak	0.2	Madurese	4.9
5	Batak	3.4	Cirebonese	3.5	Foreigners	0.1	Batak	0.3	Sundanese	0.1	Banten[h)]	3.2
6	Minangkabau	2.9	Batak	1.1	Madurese	0.04	Madurese	0.2	Other Sulawesi[k)]	0.1	Cirebonese	1.4
7	Malay	1.0	Chinese	0.8	Lampung[e)]	0.04	Minangkabau	0.2	East Nusa Tenggara[j)]	0.1	Chinese	1.1
8	Madurese	0.8	Minangkabau	0.6	Betawi	0.03	East Nusa Tenggara[j)]	0.1	Foreigners	0.1	Batak	0.8
9	South Sumatra[f)]	0.8	Malay	0.5	Malay	0.03	Dayak	0.1	Lampung[e)]	0.1	Minangkabau	0.5
10	Buginese	0.7	Lampung[e)]	0.3	Minangkabau	0.03	South Sumatra[f)]	0.1	Buginese	0.1	Malay	0.3
	Other	4.8	Other	1.6	Other	0.7	Other	1.1	Other	0.4	Other	1.4
	All	100.0	All	100.0		100.0		100.0		100.0		100.0
% of Total Population		4.0		22.6		13.6		1.5		15.7		57.5

Table A.2., continued.

Rank	BALI & NUSA TENGGARA					
	Bali (%)		West Nusa Tenggara (%)		East Nusa Tenggara (%)	
1	Balinese	86.0	Sasak	67.6	East Nusa Tenggara[j]	81.2
2	Javanese	9.6	Other West Nusa Tenggara[l]	25.8	Other Kalimantan[m]	14.5
3	Madurese	0.8	Balinese	2.7	Javanese	1.2
4	Malay	0.6	Javanese	1.8	Other Sulawesi[k]	0.9
5	Sasak	0.6	Other Sulawesi[k]	0.6	Buginese	0.5
6	East Nusa Tenggara[j]	0.5	Buginese	0.4	Other West Nusa Tenggara[l]	0.4
7	Chinese	0.4	East Nusa Tenggara[j]	0.3	Papua	0.3
8	Sundanese	0.3	Chinese	0.2	Maluku[n]	0.3
9	Buginese	0.2	Foreigners	0.1	Chinese	0.2
10	Batak	0.2	Sundanese	0.1	Balinese	0.1
	Other	0.9	Other	0.5	Other	0.5
	All	100.0		100.0		100.0
% of Total Population		1.6		1.9		2.0

Rank	KALIMANTAN									
	West Kalimantan (%)		Central Kalimantan (%)		South Kalimantan (%)		East Kalimantan[p] (%)		All Kalimantan (%)	
1	Dayak	50.0	Other Kalimantan[m]	26.7	Banjarese	74.3	Javanese	30.3	Banjarese	26.2
2	Malay[o]	18.6	Javanese	21.7	Javanese	14.5	Buginese	20.8	Dayak	21.3
3	Javanese	9.7	Banjarese	21.0	Buginese	2.0	Other Kalimantan[m]	13.4	Javanese	18.2
4	Chinese	8.2	Dayak	20.4	Dayak	1.9	Banjarese	12.5	Other Kalimantan[m]	8.1
5	Madurese	6.3	Malay[o]	3.9	Madurese	1.5	Other Sulawesi[k]	6.4	Buginese	6.9
6	Buginese	3.1	Madurese	1.9	Other Sulawesi[k]	1.4	Dayak	6.0	Malay[o]	6.6
7	Sundanese	1.1	Sundanese	1.3	Other Kalimantanm)	1.3	East Nusa Tenggaraj)	1.6	Madurese	3.0

Table A.2., continued.

Rank	KALIMANTAN, Continued									
	West Kalimantan (%)		Central Kalimantan (%)		South Kalimantan (%)		East Kalimantan[p)] (%)		All Kalimantan (%)	
7	Sundanese	1.1	Sundanese	1.3	Other Kalimantan[m)]	1.3	East Nusa Tenggara[j)]	1.6	Madurese	3.0
8	Batak	0.6	East Nusa Tenggara[j)]	0.7	Sundanese	0.7	Sundanese	1.6	Chinese	3.0
9	South Sumatra[f)]	0.5	Batak	0.6	Chinese	0.4	Madurese	1.3	Other Sulawesi[k)]	2.1
10	Banjarese	0.3	Buginese	0.4	Batak	0.3	Batak	1.1	Sundanese	1.2
	Other	1.5	Other	1.5	Other	1.8	Other	5.1	Other	3.4
	All	100.0		100.0		100.0		100.0		100.0
% of Total Population		1.9		0.9		1.5		1.5		5.8

Rank	SULAWESI									
	North Sulawesi[q)]		Central Sulawesi		South Sulawesi[r)]		Southeast Sulawesi		All Sulawesi	
1	Gorontalo	33.7	Other Sulawesi[k)]	62.2	Buginese	40.9	Other Sulawesi[k)]	62.9	Other Sulawesi[k)]	37.1
2	Minahasa	31.1	Buginese	15.6	Other Sulawesi[k)]	27.0	Buginese	22.3	Buginese	27.0
3	Other Sulawesi[k)]	28.0	Javanese	8.4	Makassarese	26.2	Javanese	7.1	Makassarese	14.4
4	Javanese	3.2	Balinese	4.4	Javanese	3.1	Makassarese	2.7	Gorontalo	7.1
5	Buginese	0.9	Gorontalo	4.0	Chinese	0.5	Balinese	2.2	Minahasa	6.9
6	Maluku[n)]	0.8	Minahasa	1.2	Balinese	0.5	Sundanese	1.1	Javanese	4.5
7	Balinese	0.6	Sasak	0.8	East Nusa Tenggara[j)]	0.4	Sasak	0.3	Balinese	1.3
8	Makassarese	0.4	Makassarese	0.7	Other Kalimantan[m)]	0.3	Maluku[n)]	0.2	Chinese	0.4
9	Chinese	0.3	Sundanese	0.6	Sasak	0.2	East Nusa Tenggara.[j)]	0.2	Sundanese	0.3
10	Foreigners	0.2	Chinese	0.5	Maluku[n)]	0.2	Other Kalimantan[m)]	0.2	Maluku[n)]	0.3
	Other	0.9	Other	1.7	Other	0.8	Other	0.7	Other	1.4
	All	100.0		100.0		100.0		100.0		100.0
% of Total Population		1.4		1.1		3.9		0.9		7.3

Table A.2., continued.

Rank	MALUKU & IRIAN JAYA				INDONESIA	
	Maluku[s)]		Irian Jaya (Papua)[t)]			
1	Maluku[n)]	70.8	Papua	71.0	Javanese	40.2
2	Other Sulawesi[k)]	19.0	Javanese	9.8	Sundanese	15.5
3	Javanese	4.8	Other Sulawesi[k)]	4.6	Batak	3.6
4	Buginese	1.8	Maluku[n]	4.6	Other Sulawesi[k)]	3.2
5	East Nusa Tenggara[j)]	0.5	Buginese	3.6	Madurese	3.0
6	Minahasa	0.5	Makassarese	1.7	Betawi	2.9
7	Papua	0.4	East Nusa Tenggara[j)]	1.2	Minangkabau	2.7
8	Makassarese	0.4	Minahasa	1.0	Buginese	2.7
9	Gorontalo	0.3	Batak	0.7	Malay	2.3
10	Sundanese	0.3	Sundanese	0.6	South Sumatra[f)]	2.2
	Other	1.3	Other	1.4	Other	21.7
	All	100.0		100.0		100.0
% of Total Population		1.1		1.5		100.0

Source: Calculated from BPS (2011)

Note: The IFLS provinces are shaded. The province names are according to the IFLS 1993 classification. a) Including Kepulauan Riau (Riau Islands province); b) Acehnese and other ethnic groups from Aceh; c) Other ethnic groups in Sumatra; d) All ethnic groups from Jambi; e) All ethnic groups from Lampung; f) Other Southern Sumatra ethnic groups; g) Including Bangka and Belitung Province; h) Bantenese and other ethnic groups from Banten Province (formerly a part of West Java province); i) Including Banten province; j) All ethnic groups from East Nusa Tenggara; k) Other ethnic groups from Sulawesi; l) Other ethnic groups from West Nusa Tenggara; m) Other ethnic groups from Kalimantan; n) All ethnic groups from Maluku; o) We assume two sub-ethnic groups of Malay in Kalimantan are originally from West Kalimantan (see Table A.1, note b); p) Including North Kalimantan province q) Including Gorontalo province; r) Including West Sulawesi province; s) Including North Maluku province; t) Including West Papua province.

Migration rates by ethnicity, Census 1930 and 2010

In this section, we show the comparison of migration rates by ethnic groups to illustrate how the mobility of these ethnic groups may have changed overtime. The migration rates of selected ethnic groups using the Census 1930 and 2010 and the ranks of these ethnic groups based on these rates, are displayed in Table A.3. The rates in 1930 taken from Naim (1973) are based on the proportions of the people belonging to a particular ethnic group who live outside their home territory (district level). We calculated the 'rates' in 2010 based on the available information in the 2010 Census publication (BPS, 2011), which is the proportion of the people belonging to a particular ethnic group who live outside their home province, regardless where they were born. The home provinces of these ethnic groups were determined by following the heartland origin of those ethnic groups from Auwalin (2020). It is difficult to determine the home province of some of the ethnic groups because their home regions could be in different provinces across an island, such as the Malay in Sumatra and the Dayak in Kalimantan.

We selected and regrouped the ethnic groups to correspond with the table from Naim (1973) using the 1930 Census. In the Census 2010 publication, the categorisation of the ethnic groups does not allow us to group them to follow precisely the ethnic groups in 1930. For example, Bawean is not available as a separate category in the Census 2010 publication. We provide two types of ranks for the Census 2010 numbers. The first rank is only for the ethnic groups available in Naim's table, while the second rank is for all the ethnic groups calculated from the 2010 Census publication.

Table A.3. Migration rates and ranks by ethnicity, Census 1930 and 2010

Ethnic group	1930		2010		
	(%)	Rank 1	(%)	Rank 1	Rank 2
Bawean[a]	35.9	1	-	-	-
Batak	15.3	2	31.7	5	6
Banjarese	14.2	3	34.9	3	3
Minangkabau	11.0	4	34.7	4	4
Buginese	10.5	5	43.3	2	2
Menado[b]	9.5	6	17.6	11	11
Ambon[c]	9.1	7	17.7	10	10
Bengkulu[a]	8.3	8	-	-	-
Mandar[a][d]	7.0	9	-	-	-
Peninsular/Coastal Malay[e]	5.2	10	30.2	6	7
Palembang[f]	4.9	11	19.5	9	9
Nias	3.7	12	12.5	13	14
Betawi[g]	-	-	60.3	1	1
Bantenese	-	-	7.2	-	19
Sundanese	-	-	15.8	-	12
Cirebonese[h]	-	-	3.4	-	24
Bantenese, Sundanese, & Cirebonese	-	-	8.6	-	-
Javanese	-	-	31.8	-	5
Madurese	-	-	9.2	-	18
Sundanese, Javanese, & Madurese[i]	3.4	13	26.4	7	-
Makassarese	2.0	14	10.9	14	15
Buginese & Makassarese[j]	8.0	-	33.7	-	-
Jambi[d]	1.8	15	5.5	17	21
Acehnese	1.1	16	6.6	16	20
Timorese[k]	0.9	17	9.4	15	17
Lampung	0.9	18	25.6	8	8
Toraja[a]	0.2	19	-	-	-
Dayak[l]	0.2	20	5.1	18	22
Sasak	0.1	21	4.4	-	23
Other West Nusa Tenggara[h]	-	-	9.7	-	16
Sasak & Other West Nusa Tenggara	-	-	5.9	-	-
Balinese	0.1	22	15.5	12	13

Source: Census 1930 and 2010 numbers were calculated from Naim (1973) for Census 1930 and BPS (2011) for Census 2010.

Note: (a) Not available as a separate group in Census 2010 publication (BPS, 2011); (b) From the number of the Minahasa (commonly called Menado by many Indonesians, consisting of several sub-ethnic groups) living outside North Sulawesi province in 2010; (c) The number in 2010 was calculated based on the number of the Maluku (Ambon is one of its sub-ethnic groups) living outside the province of Maluku and North Maluku; (d) Some mistakes are found

in the numbers in Naim's original table, which were recalculated and corrected; (e) The number in 2010 was based on the number of the Malay living outside the province of North Sumatra, Riau, Riau Island, and South Sumatra; (f) In BPS (2011), the Palembang is not presented separately. The number was then calculated for all South Sumatra ethnic groups; (g) The Betawi ethnic group was formally established after the independence and thus was not counted in 1930 (Castles, 1967); (h) Not available as a separate group in 1930; (i) Grouped as one in Naim's table; (j) The 1930 number was calculated based on the numbers for the Buginese and the Makassarese in Naim's table (k) The number in 2010 for the Timorese was calculated based on the number of all of the ethnic groups from East Nusa Tenggara province living outside their home province; (l) The number in 2010 was calculated based on the number of the Dayak living outside the province of West, Central, and North Kalimantan.

The source of information of ethnicity

We display the source of information on ethnicity in Table A.4. All respondents were pooled from the household roster of all five waves of IFLS. Migration section respondents are the respondents with migration history since age 12. 'Original respondents' refers to all migration history respondents. The final respondents used in the analysis are those who have reached at least age 36 at their final observation after dropping the missing cases of ethnicity and other characteristics.

There are two types of information on ethnicity in the IFLS: (1) Self-reported from the education section; (2) From the household roster, reported by a household representative (the head of the household or his/her spouse, or an eligible adult respondent). The responses of ethnicity in the education section are the respondent's ethnicity, the parents' ethnicity, and ethnicity that influenced the respondent's household daily life. We prioritised the self-reported answer on the question of 'where were you from' (*dari mana asal Anda*?). The respondents could identify themselves to belong to more than one ethnic group out of the 25 ethnic categories provided. We use the ethnicity of the father if one belongs to more than one ethnic group. For the matrilineal

ethnic group Minangkabau, we use the ethnicity of the mother. If parents' ethnicity is missing or the parents are also the descendants of people with mixed ethnicities, we use the ethnicity primarily influential in the respondent's household daily life. If the ethnicity is still missing, we impute the ethnicity from the roster or of the immediate family members (parents, children, siblings). We exclude the rest of the respondents if their ethnicity was still missing.

Auwalin (2020) used the daily language at home to estimate missing ethnic information. We prioritised using the ethnicity of the immediate family members to estimate the missing ethnic information because around 13 per cent of the IFLS respondents speak only the national language, Indonesian, at home. According to Census 2010, 19.9 per cent of Indonesians speak Indonesian at home (BPS, 2011). Among the respondents, 98 per cent of their ethnicity is from the education section, and 0.6 per cent is from the roster.

Table A.4. Distribution of respondents by the source of information for ethnicity

Source of information	All respondents	Migration section respondents		
		Original Respondents	Final respondents	
			All	Migrants
	(%)	(%)	(%)	(%)
Education section	53.41	94.01	97.76	98.08
Roster section	34.84	0.57	0.63	0.55
Imputed with the ethnicity of:				
Father/head of household	2.88	0.65	0.21	0.12
Mother/spouse of the head of household	1.22	0.40	0.13	0.11
Child	1.07	0.77	1.27	1.12
Sibling	0.07	0.02	0.01	0.02
Missing	6.51	3.59	-	-
Total	100	100	100	100
Observations	83333	46626	21337	12161

Source: Author’s calculation, 1993, 1997, 2000, 2007, and 2014 IFLS.

Several key characteristics of respondents and migrants by ethnicity

In Table A.5, we show substantial differences between the ethnic groups for several key characteristics. Firstly, migrants are a much younger subset than all respondents. Furthermore, there are substantial differences in the composition of the ethnic groups according to birth cohort. For example, the proportion of the younger cohort among the Balinese migrants is much higher than among all the Balinese. In contrast, for the Betawi, the proportions of the younger cohort for all respondents and only migrants are sr. The difference in the distribution by cohort suggests that the age dimension does not work in the same way amongst all groups but is relevant to control.

Migration is selective according to education for all ethnic groups. However, for some groups, the migrants deviate further from the general group than others. The statistics also suggest that, for the Balinese, for example, there is an intergenerational leap in terms of parents' education and their children's. They have a relatively low proportion of parents' years of schooling longer than six years, but the proportion of Balinese who finished high school is among the highest. The fact that the proportion of Balinese migrants who moved for education is only second to the Batak migrants' is in line with this. Unsurprisingly, the Betawi have the lowest proportion of those in the villages at the beginning of observation as they are originally from Jakarta, which has been categorised as urban areas for decades. We can see that the Minangkabau and the Banjarese have the highest proportions of those who stayed for a shorter length after the first migration. In contrast, the Sasak, Bima, and Dompu have the lowest proportion of staying for a shorter length.

Table A.5. The percentage of birth cohort 1970 and later, parent's years of schooling (YoS) more than six years, respondents who finished at least high school, living in the village at age 12, first migration for work, education, and marriage, length of stay up to two years for all respondents and migrants, by ethnicity

Ethnicity	Cohort ≥1970		YoS >6 years		≥High School		In village at age 12		First migration's reason[a)]				Stay
	All (%)	Migrants (%)	All (%)	Migrants (%)	All (%)	Migrants (%)	All (%)	Migrants (%)	Work (%)	Education (%)	Marriage (%)	Family (%)	≤2 years (%)
Batak	29.4	34.1	24.9	27.7	39.4	46.3	70.7	68.1	25.7	17.5	20.6	24.6	18.9
Minangkabau	29.6	33.9	20.4	25.2	36.7	46.7	69.8	65.3	27.1	14.4	16.1	30.3	23.5
Malay & South Sumatra	32.3	36.0	20.0	23.4	32.5	37.3	74.3	71.9	18.8	13.2	24.2	30.9	17.9
Bantenese, Sundanese, & Cirebonese	28.3	31.5	15.7	19.5	25.9	33.6	68.0	62.5	22.5	6.5	31.3	24.6	19.0
Betawi	31.2	31.8	16.2	18.5	31.2	36.5	38.0	31.8	11.9	2.4[b)]	27.5	27.0	18.3
Javanese	28.1	32.2	13.6	18.0	26.0	34.2	73.8	69.2	28.8	8.7	28.1	22.1	20.9
Madurese	23.2	37.9	6.3	9.6	12.2	17.5	73.6	66.3	28.3	5.0[c)]	35.4	24.2[d)]	16.3
Banjarese	30.5	32.2	13.1	17.5	24.9	32.7	72.0	65.9	19.2	9.0	27.5	30.3	22.8
Buginese & Makassarese	30.1	35.7	8.6	12.6	19.7	28.8	76.6	71.6	17.8	11.7	26.5	31.6	20.6
Balinese	30.7	40.9	8.9	15.3	30.6	46.2	81.0	73.9	31.0	17.1	23.9	17.4	18.3
Sasak, Bima & Dompu	32.2	35.7	8.1	11.4	18.9	26.7	89.6	85.6	16.2	16.0	33.1	20.4	13.6
Others	18.1	20.1	19.8	26.6	30.8	40.3	59.7	49.0	25.4	12.8	21.0	21.0	21.2

Source: Authors' calculation using all respondents with migration history since age 12 up to at least 36 years of 1993, 1997, 2000, 2007, 2014 IFLS.

Note: The Pearson χ^2 tests of independence show that all variables are significantly related to ethnicity at the five-per cent level. (a) Row percentage of all reasons by ethnicity. The rest of the reasons are not presented. The Pearson χ^2 tests were calculated based on (b) 9 cases for the Betawi, (c) 12 cases and (d) 17 cases for the Madurese.
